# Silicon fertigation alleviates salinity stress by enhancing morpho-physiological, photosynthetic, antioxidative responses, and yield in mung bean (*Vigna radiata* L.) varieties Co7(Gg) and Co8 under pot and field conditions

**DOI:** 10.3389/fpls.2025.1693710

**Published:** 2025-12-02

**Authors:** Sushilkumar Sadhanandan, Dong Won Bae, Muthu Arjuna Samy Prakash, Sowbiya Muneer

**Affiliations:** 1 Department of Horticulture and Food Science, School of Agricultural Innovations and Advanced Learning, Vellore Institute of Technology, Vellore, India; 2 Department of Genetics and Plant Breeding, School of Agricultural Innovations and Advanced Learning, Vellore Institute of Technology, Vellore, India; 3 Central Instrument Facility, Gyeongsang National University, Jinju, Republic of Korea; 4 Department of Genetics and Plant Breeding, Faculty of Agriculture, Annamalai University, Annamalai Nagar, Tamil Nadu, India

**Keywords:** abiotic stress, isozymes, Si deposition, thylakoidal complex proteins, yield attributes

## Abstract

**Introduction:**

Silicon (Si), the second most prevalent element in the earth, is not soluble enough for plants to absorb, despite being one of numerous helpful elements. Supplementing with silicon (Si) is seen to be one of the most promising ways to mitigate abiotic stressors like salt and drought. Legume crops are still underutilised, especially mung bean, an important crop in India, despite several research on the effects of salt and silicon stress on various crops.

**Methods:**

In order to study the impact of exogenous application of Si concentrations on the growth and physiochemical, Photosynthetic efficiency, and antioxidative pathways of four mung bean cultivars—K1, Co6, Co7Gg, and Co8 exposed to two levels of salinity] [10 mM NaCl, 20mM NaCl and 5 mM of Si—a Pot experiment and field data were collected over the course of two growth seasons (2023–2024). The overall treatments given were in six combinations as: (i) -Si/-NaCl (control) (ii) -NaCl + Si (iii) 10mM NaCl/−Si (IV). 10 mM NaCl/+ Si (v) 20mM NaCl/-Si (vi) = 20mM NaCl/+Si Growth factors like biomass, plant length, height, and photosynthetic measurements were all lowered by salinity stress; however, these effects were lessened by silicon supplementation at a concentration of 5 mM sodium silicate.

**Results and discussion:**

Under salt stress, the presence of silicon boosted the production of photosynthetic proteins such Photosystem I, Photosystem II, and light harvesting complexes. In the mung bean, salinity stress also resulted in oxidative damage in the form of superoxide radical (O2−) and hydrogen peroxide (H2O2), which raised MDA (lipid peroxidation) and electrolyte leakage. On the other hand, 5 mM sodium silicate has the ability to scavenge free radicals, which lowers electrolyte loss and lipid peroxidation (MDA). Significant silica deposition in the leaf epidermis was associated with this, and in the end, this served as a mechanical barrier to lessen the harmful effects of salt stress. Our simulated investigations, which employed Si as a supplement, indicated that, of all the varieties used in this study, K1 and Co7Gg shown resilience to salt stress, but Co6 and Co8 showed sensitivity. Experiments conducted at the field level on yield and growth provided evidence for the study's findings that Si has a positive impact on salt stress. Si reduced the detrimental effects of salt stress and provided a basic idea for using Si as a fertilizer.

## Introduction

Biotic and abiotic stress reduce agricultural output globally; however, abiotic stresses specifically influence the spread of plant species within various environmental zones (Chaves et al., 2009; Gong et al., 2020; Godfray et al., 2010). Soils are under increasing strain due to unsustainable agricultural methods, overexploitation of natural resources, and population growth, which is leading to worrying rates of soil degradation worldwide. According to estimates, over 833 million hectares of cultivable soil globally have already been affected by salt, covering more than 10% of agricultural land. This represents a significant risk to global food security. Central Asia, the Middle East, South America, North Africa, and the Pacific are among the areas most severely impacted (Chaves et al., 2009). Salinity stress has affected 6.73 million hectares of land (4.2% of total agricultural land) in India, causing yield losses greater than 60%. At the cellular level, salt stress leads to the formation of reactive oxygen species (ROS), despite activation of salt overlay sensitive (SOS) genes and antioxidative enzymes. Some plant species, however, are susceptible and cannot effectively mitigate the damage caused by salinity.

To overcome salinity stress in plants, several agricultural practices such as gypsum application are in use, especially in alkaline soils. Recently, the application of beneficial elements through fertigation has emerged as a promising strategy, with silicon (Si) being particularly noteworthy. Si is classified as a beneficial or quasi-essential mineral element; its absorption is enhanced under salt stress and depends on soil type and pH (Liang et al., 2005, 2018; Shah and Wu, 2019). Silicon uptake is an active process, and crops like rice can accumulate over 1% Si to improve transpiration (Ma, 2004; He et al., 2013, 2015). Si strengthens plant mechanical structure, reduces lodging, and promotes development and productivity under both biotic and abiotic stresses (Ma and Yamaji, 2006; Zhu and Gong, 2014; Exely, 2015; Thakral et al., 2021; Guerriero et al., 2016). It also enhances defense-related signaling and maintains physiological and biochemical functions (Ye et al., 2013; Manivannan and Ahn, 2017; Al Murad and Muneer, 2023; Costa et al., 2024; Yoshiyuki and Asada, 1981). Si deposition in plants can improve salinity tolerance by increasing the thickness of the Casparian strip and suberin lamellae, thereby limiting sodium ion movement from roots to shoots (Yeo, 1999; Luyckx et al., 2017; Al Murad and Muneer, 2022).

The inclusion of legumes in cropping systems contributes significantly to agricultural sustainability (Arnoldi et al., 2014; Araújo et al., 2014; Stagnari et al., 2017). Legumes are essential for food and feed globally due to their rich nutritional value and their ability to fix atmospheric nitrogen through symbiosis with rhizobia. However, legumes are particularly sensitive to abiotic stress, especially in arid and semi-arid regions where evapotranspiration exceeds rainfall and where salinity is high due to elevated Na^+^ and Cl^-^ concentrations in the root zone. Mung bean (Vigna radiata L.), an important legume grown across 6 million hectares globally, is particularly valued for its short growth cycle (65–90 days), adaptability, and minimal input requirements (Nair et al., 2012; Singh et al., 2007). Despite its nutritional richness, mung bean is often cultivated on marginal, low-input lands, making it vulnerable to stress (Nair et al., 2012). It fits well into existing crop rotations in India and other regions, but yields are still severely affected by salinity stress—especially in southern Indian regions using high electrical conductivity irrigation water.

The vulnerability of mung bean to both biotic and abiotic stresses has been well documented (Sehrawat et al., 2013). Globally, salinity causes yield losses exceeding 60%, and projections suggest that by the mid-21st century, over 50% of arable land will be salinized (Hasanuzzaman et al., 2012). Despite various soil reclamation efforts, many developing countries like India have not adopted these measures due to their high cost. An alternative, more economical strategy is the use of silicon supplementation to mitigate salt stress (Borawska-Jarmułowicz et al., 2022; Putra et al., 2022). Our prior work has shown that Si can enhance mung bean yield in alkaline soils and regulate stress-responsive proteins under salt stress (Al Murad and Muneer, 2022, 2023).

Si mimics carbon in plants and interacts with biomolecules such as nucleic acids and structural proteins. Legumes are widely consumed in India but are often grown in saline, alkaline, and calcareous soils—especially in Tamil Nadu, which ranks fourth among Indian states for saline soils. Although pest-resistant legume varieties are available in Tamil Nadu, few have been evaluated for salinity tolerance. Conventional approaches to mitigate salinity stress are often costly or impractical for smallholder farmers. Given these gaps, this study aimed to evaluate the effect of Si fertigation on morpho-physiology, photosynthesis, and antioxidative mechanisms in four widely grown mung bean varieties from Tamil Nadu (K1, Co6, Co7(Gg), and Co8). The varieties were also analyzed for yield performance under salt stress in field conditions.

## Material and methods

### Pot culture

Seeds of four mung bean (*Vigna radiata* L.) cultivars—K1, Co6, Co7(Gg), and Co8—were procured from Tamil Nadu Agricultural University, Tamil Nadu, India. Before sowing, seeds were disinfected by immersion in 1% (v/v) sodium hypochlorite for 2 min and then rinsed thoroughly with distilled water. The experiment followed a completely randomized block design, with seeds sown in pots filled with a sterilized mixture of red soil, sand, and vermicompost in equal proportions (1:1:1). Pots (13 cm diameter × 17 cm height) contained approximately 900 g of the soil mix and were maintained in a polyhouse at the School of Agricultural Innovations and Advanced Learning (VAIAL), Vellore Institute of Technology, India. Temperature inside the polyhouse was regulated between 27–30 °C, relative humidity maintained at 65% (monitored using a Richie HTC infrared/optical digital thermometer), and photosynthetically active radiation (PAR) kept within 400–700 nm.

**Figure 1:**
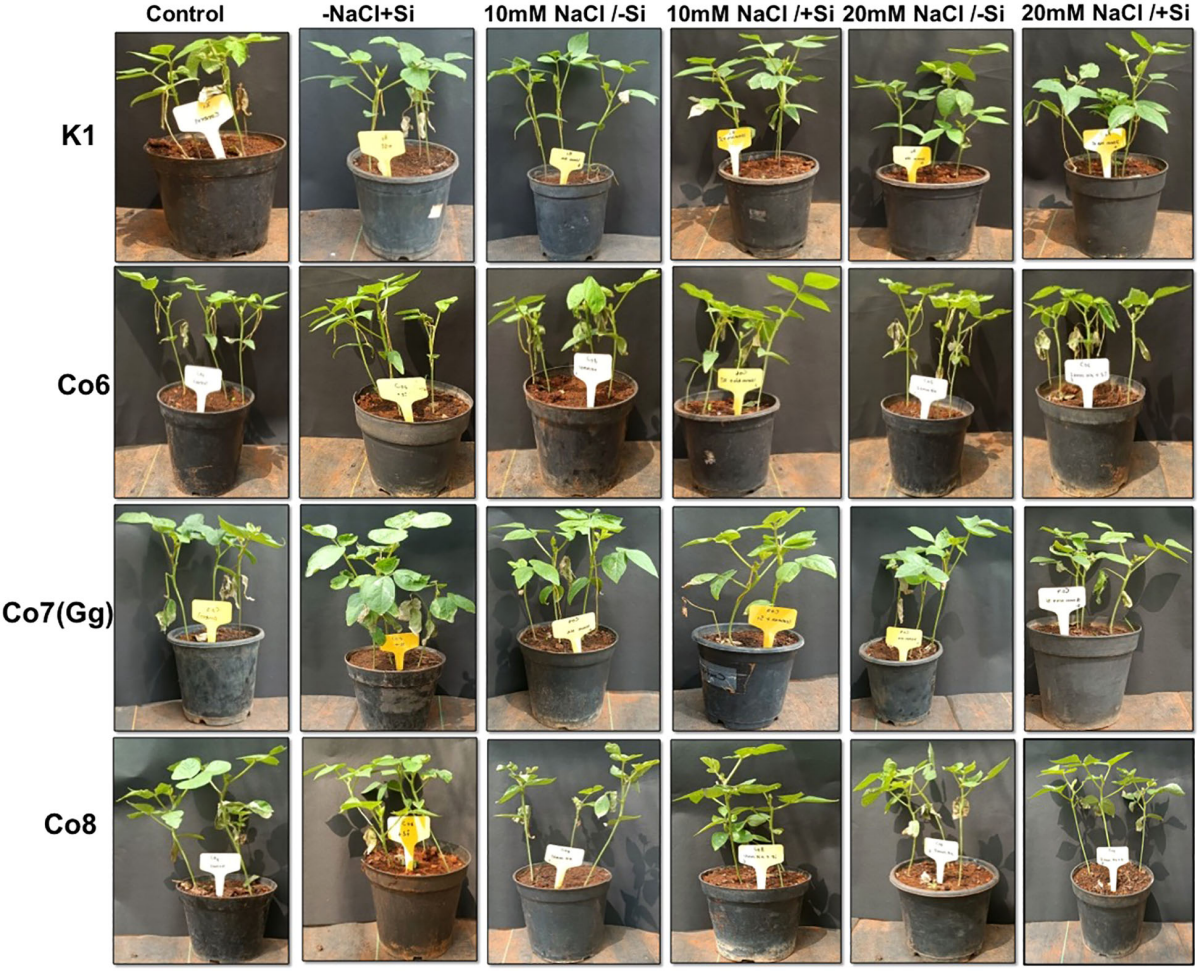
Morphological representation of pot culture of mung bean varieties (*Vigna radiata L.*); K1, Co6, Co7(Gg), and Co8 under 5 mM Si supply and salinity stress after six treatments, (i) Control, (ii) -NaCl+Si (iii) 10 mM NaCl/-Si (iv) 10 mM NaCl/+ Si (v) 20 mM NaCl/–Si (vi) 20 mM NaCl/+Si for a period of 10 days.

After germination, seedlings were thinned to four per pot. At 30 days after sowing (DAS), plants were allocated randomly to salinity and silicon treatments. Silicon (Si) was supplied as 5 mM sodium silicate according to a previously standardized protocol (Al Murad and Muneer, 2022). Six treatment combinations were applied: (i) –Si/–NaCl (control), (ii) –NaCl + Si, (iii) 10 mM NaCl/–Si, (iv) 10 mM NaCl/+Si, (v) 20 mM NaCl/–Si, and (vi) 20 mM NaCl/+Si (**Figure 1**). In total, 96 pots were used (6 treatments × 4 varieties × 4 replicates). Silicon was applied through soil drenching/sub-irrigation at the crop’s critical growth stage, while salinity stress was induced with water made with the specified NaCl concentrations. Soil pH (~5.6) and electrical conductivity (~0.68 mS/cm) were monitored every alternate day. Leaf samples were harvested on the 5th and 10th day after treatment, immediately frozen at –80 °C, and later used for biochemical and molecular assays. Physiological traits—photosynthesis, transpiration rate, stomatal conductance, and PSII quantum yield—were measured using portable instruments under polyhouse conditions. All experiments complied with institutional and national guidelines.

### Field preparation/yield assessment

Field trials were carried out in two season - pre-kharif season (February–May 2024) and Kharif season (June-September 2024) in the Mini Orchard and Peral Research Park Agricultural Farm respectively in School of Agricultural Innovations and Advanced Learning, covering 1152 m². All experiments were conducted following the prevailing agrometeorological conditions, including temperature, humidity, and other climatic parameters (**Supplementary Data**). The experiment followed a factorial randomized block design (FRBD) with sufficient degrees of freedom (n > 15). The soil at the site was red in texture, with pH 6.9, EC 0.68 mS/cm, organic carbon 0.83%, and nutrient content of N—188.16 kg/ha, P—1.60 kg/ha, and K—171.86 kg/ha. Six treatments identical to the pot experiment were imposed in triplicate (6 × 4 varieties × 3 replicates = 72 plots). Varieties were coded as V1—K1, V2—Co6, V3—Co7(Gg), and V4—Co8 (**Supplementary Figure 1**). Seeds, treated with *Trichoderma viride* was applied to all treatments uniformly as biocontrol agent of mung bean as a seed treatment before the onset of the *pre-Kharif* season in Tamil Nadu (March–May) under warm soils, rains, and residual salinity conditions conducive to soilborne pathogens like Fusarium spp., *Rhizoctonia bataticola*, and Pythium spp. The use of *T. viride* as a preventive biocontrol in the region and is also agronomic practices to promote consistent seedling establishment. Seeds were sown by dibbling in 4 × 4 m plots with 30 × 10 cm spacing. Irrigation was provided by flood method every 10 days, and weeds were removed manually every 15 days. The irrigation water had pH 7.5, EC 2.6 mS/cm, and total dissolved solids (TDS) of 1190 mg/L. Germination occurred between 5–7 DAS, and plants were allowed to grow for 30 days before imposing treatments. In the agronomic experiment, silicon (Si) and NaCl solutions each at a concentration of 5 mM, were applied via soil drenching during a crops critical stage of growth. Each plot (140 mung bean plants) received 14 liters of solution (equivalent to 100 mL of 5mM sodium silicate for Si treatment). All treatments were applied at the early flowering stage of development, which is considered a critical physiological stage of growth for mung bean plants. The timing was selected to compare the effects of silicon supplementation on salinity stress during a period of sensitive developmental changes. During the 10-days treatment period, irrigation was withheld to avoid leaching while maintaining adequate moisture to prevent drought stress. Post-treatment, irrigation returned to the original schedule. Initial post-treatment measurements included plant height, biomass, and SPAD chlorophyll readings. Plants grew to full maturity (~90 DAS), after which yield-related parameters (number of flowers, flower abortion, pod length, pods per plant, seeds per 100 pods) were recorded. Integrated pest and disease management (IPDM) strategies—border cropping and botanical extracts—were implemented.

### Histochemical detection and silica bodies

Silica body staining was carried out according to Yokoyama et al. (2009). Fully Opened third leaf top of the plant were collected from each plant, were harvested from each of the biologically independent plants. The leaves were fixed at room temperature for 24h in FAA solution (formaldehyde:80% ethanol:glacial acetic acid, 90:5:5). The fixed portions underwent ethanol dehydration (80%, 90%, and 100%, 20 min each), followed by benzene-ethanol washes of increasing benzene concentration (10–100%, 20 min each). Benzene-equilibrated samples were stained in 0.1% crystal violet lactone (in benzene) and observed under a phase contrast microscope (MT4300L, MEIJI TECHNO CO., Japan).

### Gas exchange and PSII quantum yield

Transpiration rate, stomatal conductance, and net photosynthetic rate were determined from SPAD readings (Konica Minolta, Tokyo, Japan) using the following equations:


$$NPR\;=\;0.0049x^{2}\;+\;0.0383x\;-\;4.1593$$

$$SC\;=\;0.0027x^{2}\;+\;0.00214x\;+\;0.0808$$

$$T\;=\;0.0094x^{2}\;+\;0.1627x\;+\;1.0563$$

where NPR = net photosynthetic rate, SC = stomatal conductance, T = transpiration rate, and x = SPAD value.

Chlorophyll fluorescence (Fv/Fm) was measured using a mini-PAM 2000 fluorometer (Heinz Walz GmbH). Leaves were dark-adapted for 30 min before readings. The maximum quantum efficiency of PSII was calculated as Fv/Fm = (Fm − F0)/Fm (Al Murad and Muneer, 2022).

### Relative water content (RWC %)

RWC was determined following Turner (1981). Fresh leaves were weighed (FW), floated in distilled water for 4 h at 25 °C to obtain turgid weight (TW), and then oven-dried at 80 °C for 24 h to record dry weight (DW). RWC (%) was calculated as:


RWC (%) = [(W−DW)/(TW−DW)] x 100

Where fresh weight (FW); turgid weight (TW); dry weight (DW).

### Lipid peroxidation level (MDA content)

MDA levels were estimated as per Heath and Packer (1968). Leaf tissue (0.5 g) was homogenized in 0.1% TCA, centrifuged (7000 rpm, 10 min), and mixed with 0.5% TBA in 20% TCA. After heating at 95 °C for 30 min, samples were cooled on ice, and absorbance was measured at 532 nm with 600 nm readings subtracted for turbidity correction.

### Localization of oxidative stress markers (H_2_O_2_ and O_2_^-^)

H_2_O_2_ was detected by vacuum-infiltrating leaves in 0.1% DAB (pH 6.5) for 5 min and incubating in darkness for 12 h. Samples were boiled in 95% ethanol to remove chlorophyll, and brown precipitates were imaged.

For O_2_^-^, leaves were incubated in 0.1% NBT in K-phosphate buffer (pH 6.4) with 10 mM sodium azide and processed similarly. Blue formazan deposits indicated superoxide presence.

### Estimation of antioxidants enzyme activity and their relative staining

Enzymes were extracted in phosphate buffer (50 mM, pH 7.0) containing EDTA, Triton X-100, and PVP. SOD activity was assayed using the NBT photoreduction inhibition method (Giannopolitis and Ries, 1977), CAT activity by Manivannan et al. (2017), and APX activity following Al Murad and Muneer (2022). Native PAGE was used for isozyme staining of APX, CAT, and SOD with specific staining protocols.

### Protein extraction and one dimensional gel electrophoresis (SDS-PAGE)

Proteins were extracted using Tris-HCl buffer (40 mM, pH 7.5) with additives (EDTA, β-mercaptoethanol, PVP, Triton X-100) as per Muneer et al. (2014). After centrifugation (13,000 rpm, 4 °C, 10 min), supernatants were quantified (Bradford assay), resolved on 12.5% SDS-PAGE gels, and stained with Coomassie Brilliant Blue.

### Thylakoid multiprotein complex analysis (1D-BN-PAGE)

Thylakoid multiprotein complexes were analyzed using blue native PAGE (BN-PAGE) following the protocol of Muneer et al., 2014 with minor modifications. Fresh leaf tissue (2 g) was homogenized in liquid nitrogen and suspended in extraction buffer (pH 7.8) containing 20 mM Tricine-NaOH, 70 mM sucrose, and 5 mM MgCl_2_. The homogenate was filtered through Mira cloth and centrifuged at 4,500 rpm for 10 min at 4 °C. The resulting pellet was resuspended in the same buffer and recentrifuged under the same conditions. The final thylakoid membrane pellet was washed with a pH 7.0 solution containing 50 mM Bis-Tris-HCl, 330 mM sorbitol, and 0.1 mg mL^-^¹ pefabloc. For solubilization, an equal volume of resuspension buffer and solubilization buffer was added to the pellet, and the mixture was kept on ice for 2–3 min. Insoluble material was removed by centrifugation at 18,000 rpm for 15 min at 4 °C. The supernatant was mixed with loading dye (0.1% CBB-G250, 100 mM Bis-Tris-HCl pH 7.0, 30% w/v sucrose, and 500 mM ϵ-amino-n-caproic acid), quantified, and loaded onto a 5–12.5% acrylamide gradient gel. Electrophoresis was initially run at 70 V for 15–20 min and then increased to 120 V until completion of the separation.

### Yield data collection

Data were recorded manually for each treatment × variety combination. Measured parameters included plant height, biomass, flower number, pod number, seeds per 100 pods, and SPAD chlorophyll (n = 15 for each).

### Statistical analysis

Statistical analysis was carried out using JMP PRO 17 (SAS Institute Inc., Cary, NC, USA). A two-way ANOVA was applied for four biological replicates, with significance set at p < 0.05. Data are expressed as mean ± SE.

## Results

### Morphological changes

Morphological alterations were the primary modifications seen in all mung bean varieties under salt stress (**Figure 1**). There was a noticeable difference in the morphology of every variety of mung bean, including fewer and yellow leaves. We found that plants treated with Si alone, especially those with Co7(Gg) and Co8, showed a notable variance. Furthermore, a considerably lower quantity of leaves was found at 20mM NaCl/-Si in comparison to both the control and 10mM NaCl/-Si. On the other hand, more leaves with a greener color were seen when Si was given. Overall, it was shown that Co7(Gg) and Co8 fared better when there was an abundance of Si than when there was a salinity stress.

**Figure 2:**
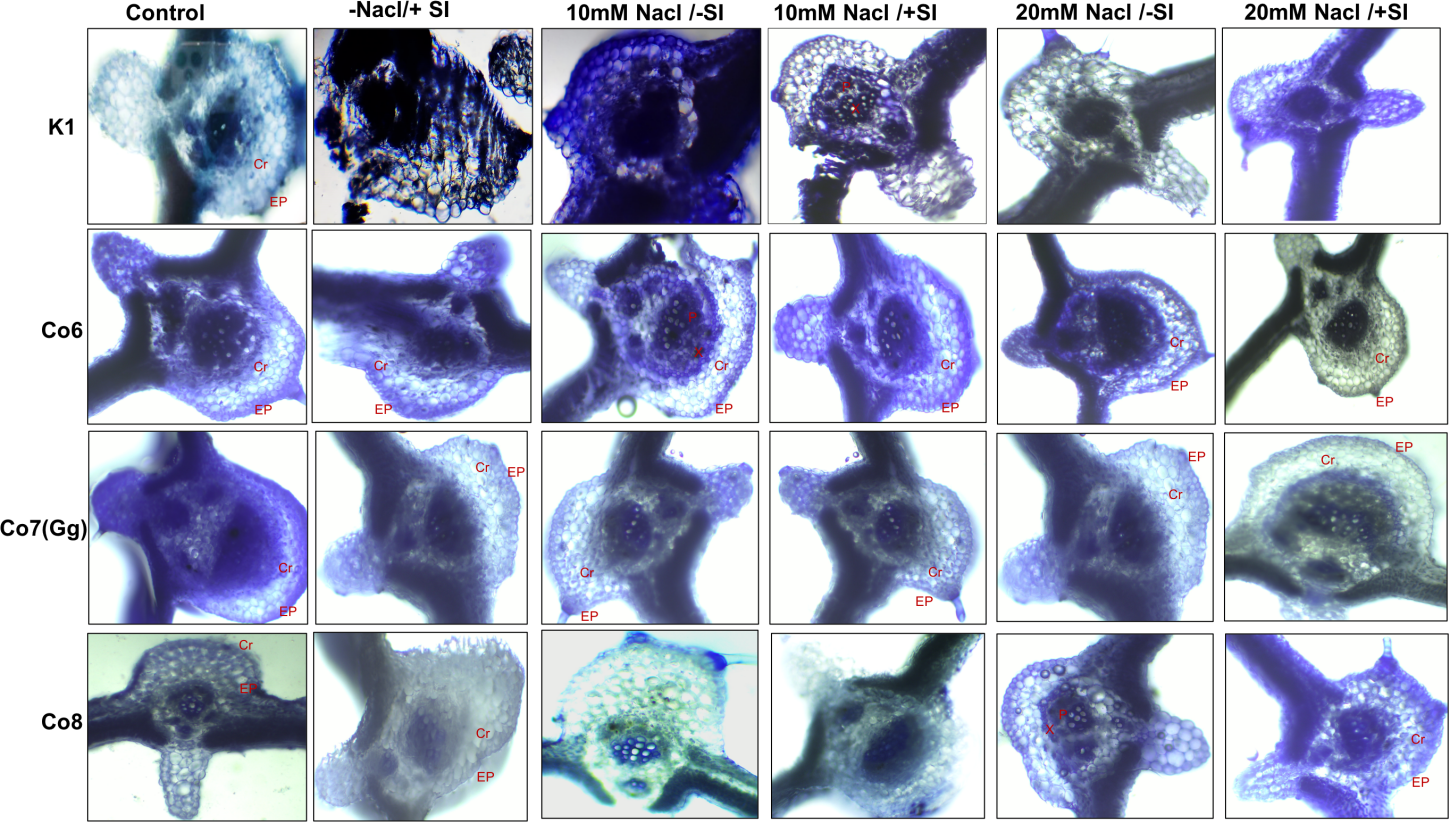
Silica deposition in mung bean varieties (*Vigna radiata L.*); K1, Co6, Co7(Gg), and Co8 under 5 mM Si supply and salinity stress after six treatments, (i) Control, (ii) -NaCl+Si (iii) 10 mM NaCl/-Si (iv) 10 mM NaCl/+ Si (v) 20 mM NaCl/–Si (vi) 20 mM NaCl/+Si for a period of 10 days. Ep indicates epidermis; Cr indicates cortical region, X indicates xylem and P indicates phloem.

Silica deposition studies were used to examine Si deposition because morphologically plants showed that Si is improving the salt stress conditions in mung bean varieties (**Figure 2**). The silica staining revealed that Si was effectively deposited in the cortical, and epidermal layers of leaves, with Co7(Gg) and Co8 exhibiting the highest levels of deposit. When Si was given, silica deposition enhanced under salinity stress (20 mM NaCl/-Si), but it was restricted when K1 and Co6 varieties were used (**Figure 2**). The silica deposition experimental analysis showed that the soaking method’s supply of silicon had effectively permeated plant tissues.

### Photosynthesis

**Figure 3:**
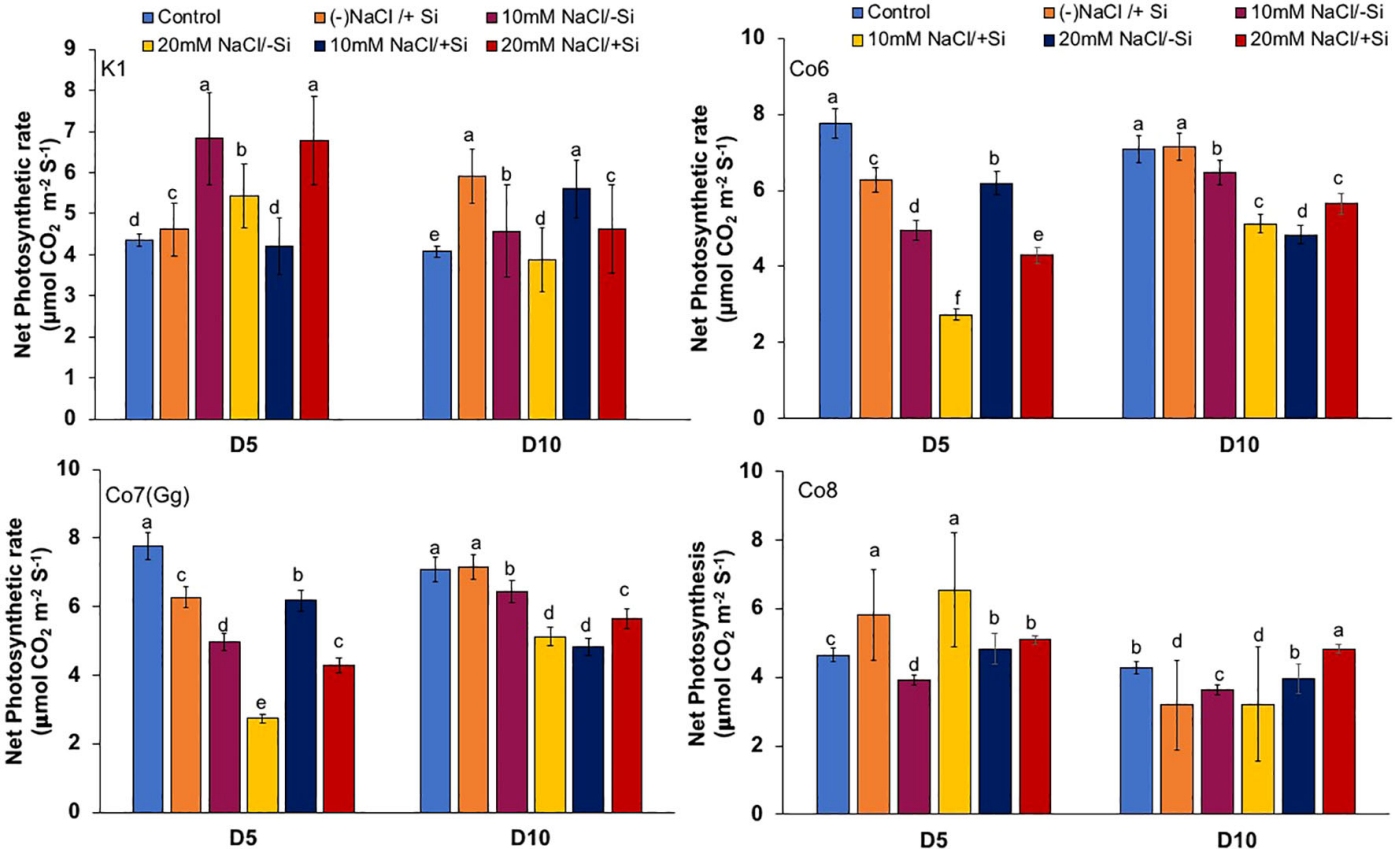
Changes in net-photosynthetic rate in mung bean varieties (*Vigna radiata L.*); K1, Co6, Co7(Gg), and Co8 under 5 mM aSi supply and salinity stress after six treatments, (i) Control, (ii) -NaCl+Si (iii) 10 mM NaCl/-Si (iv) 10 mM NaCl/+ Si (v) 20 mM NaCl/–Si (vi) 20 mM NaCl/+Si for a period of 10 days. Vertical bars indicate Mean ± SE of the means for n = 4. Means denoted by the different letter are significantly different at P ≤ 0.05.

**Figure 4:**
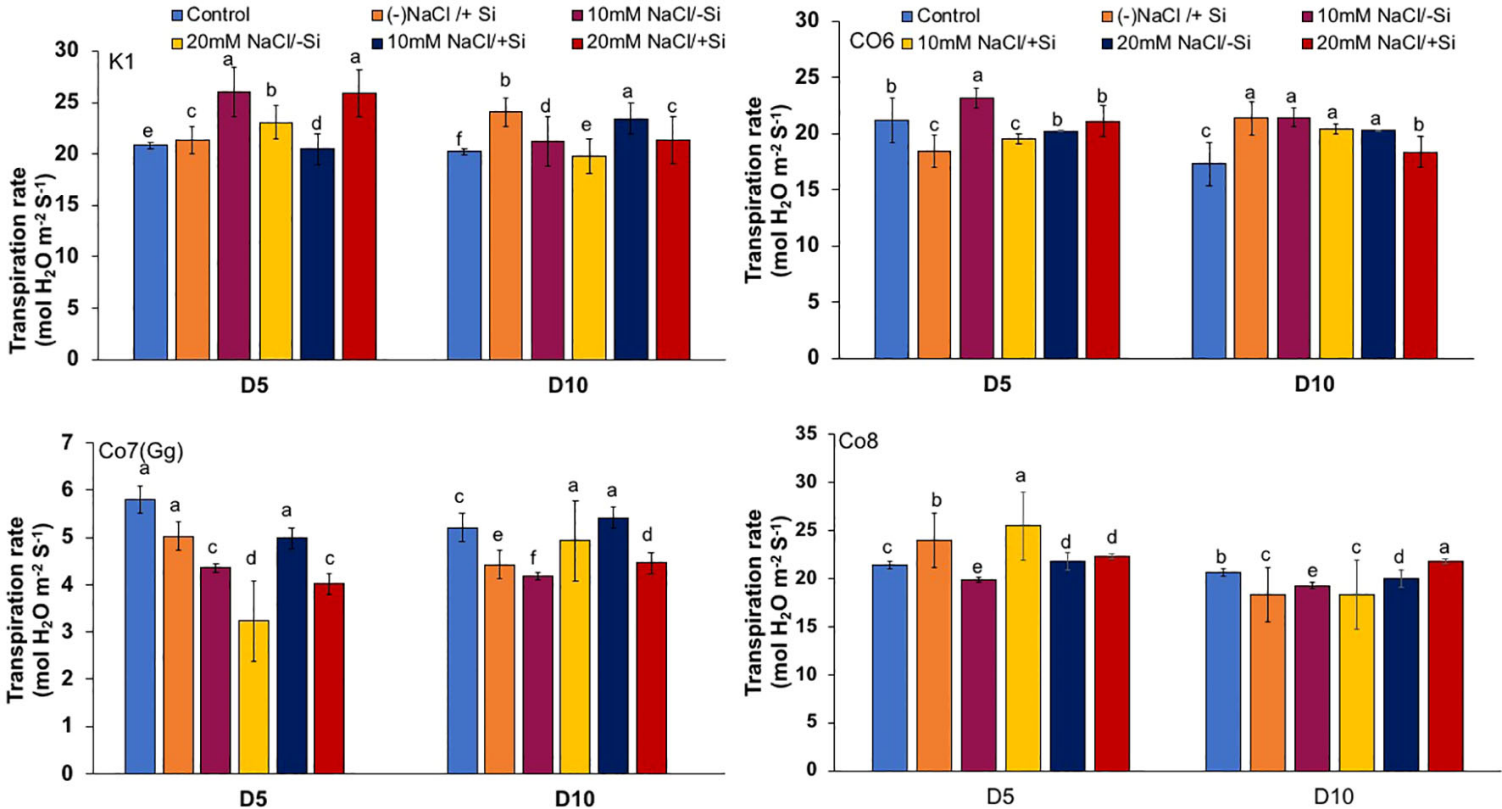
Changes in transpiration rate in mung bean varieties (*Vigna radiata L.*); K1, Co6, Co7(Gg), and Co8 under 5 mM Si supply and salinity stress after six treatments, (i) Control, (ii) -NaCl+Si (iii) 10 mM NaCl/-Si (iv) 10 mM NaCl/+ Si (v) 20 mM NaCl/–Si (vi) 20 mM NaCl/+Si for a period of 10 days. Vertical bars indicate Mean ± SE of the means for n = 4. Means denoted by the different letter are significantly different at P ≤ 0.05.

**Figure 5:**
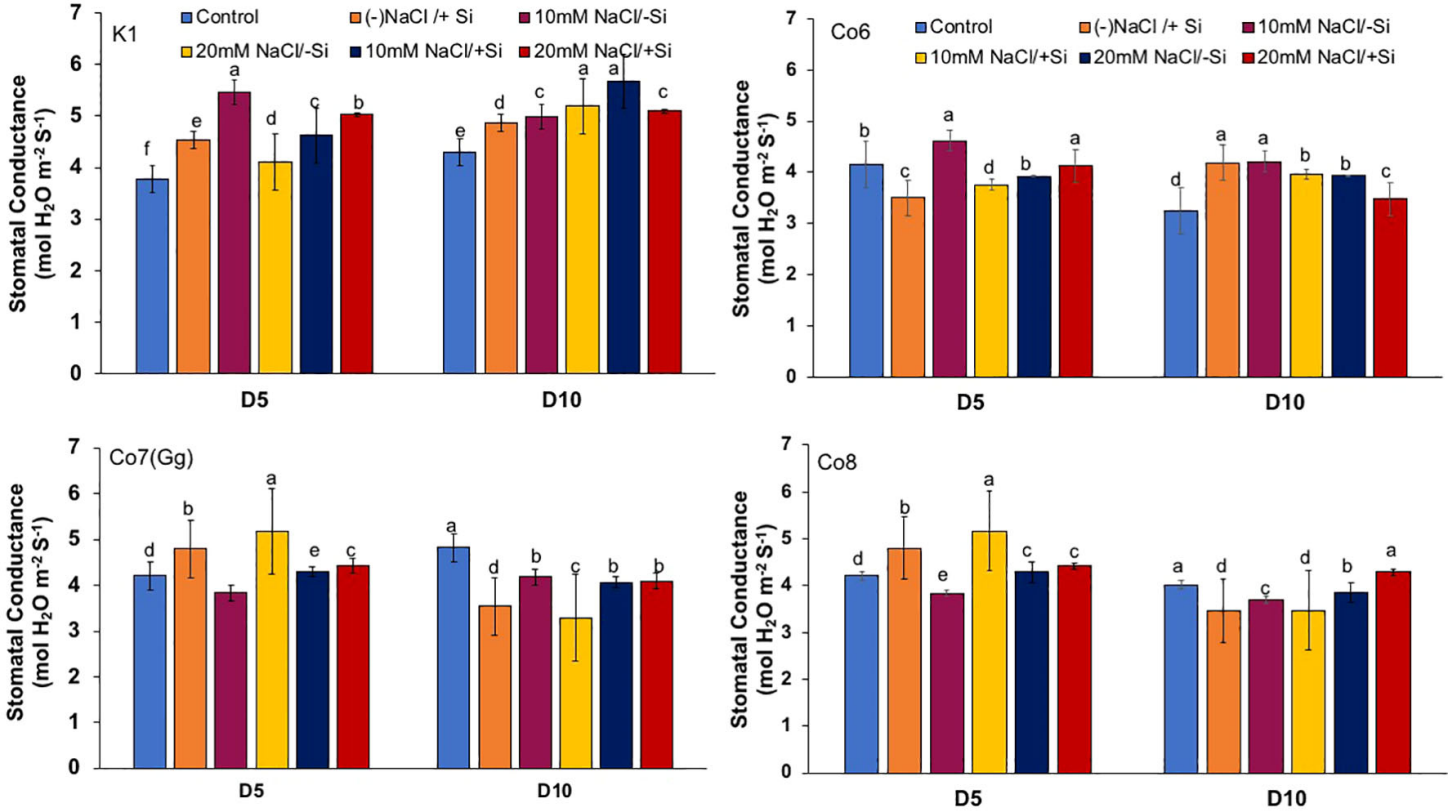
Changes in stomatal conductance in mung bean varieties (*Vigna radiata L.*); K1, Co6, Co7(Gg), and Co8 under 5 mM Si supply and salinity stress after six treatments, (i) Control, (ii) -NaCl+Si (iii) 10 mM NaCl/-Si (iv) 10 mM NaCl/+ Si (v) 20 mM NaCl/–Si (vi) 20 mM NaCl/+Si for a period of 10 days. Vertical bars indicate Mean ± SE of the means for n = 4. Means denoted by the different letter are significantly different at P ≤ 0.05.

**Figure 6:**
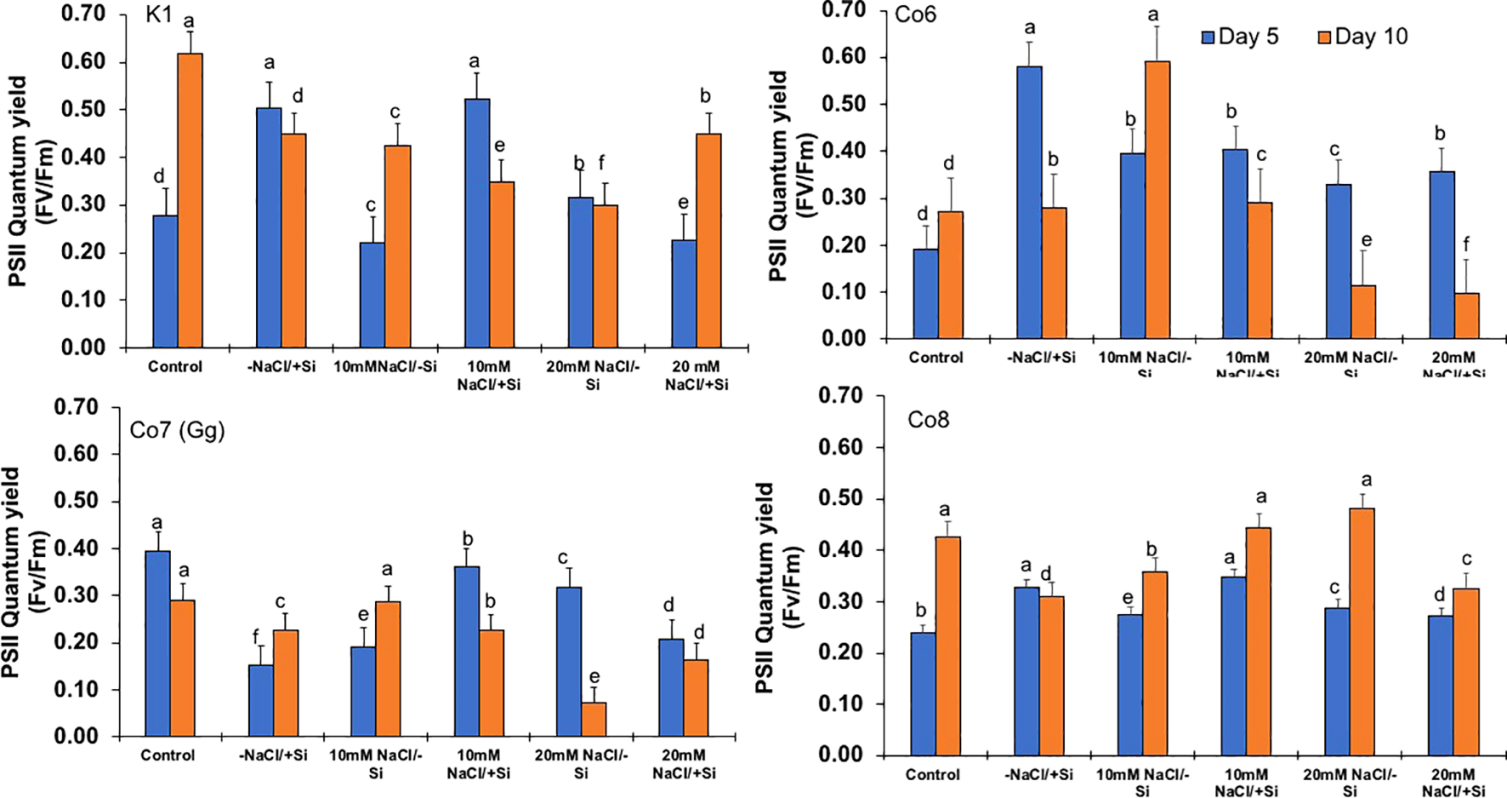
Changes in PSII quantum yield rate in mung bean varieties (*Vigna radiata L.*); K1, Co6, Co7(Gg), and Co8 under 5 mM Si supply and salinity stress after six treatments, (i) Control, (ii) -NaCl+Si (iii) 10 mM NaCl/-Si (iv) 10 mM NaCl/+ Si (v) 20 mM NaCl/–Si (vi) 20 mM NaCl/+Si for a period of 10 days. Vertical bars indicate Mean ± SE of the means for n = 4. Means denoted by the different letter are significantly different at P ≤ 0.05.

The two most important physiological parameters for a plant’s life are gaseous exchange and photosynthesis, and it is clear that both parameters may be modulated by climatic conditions or any other type of stress. In mung bean varieties, we measured four key parameters: net-photosynthesis, transpiration, stomatal conductance, and quantum yield of PSII (**Figures 3**–**6**). We found that all the photosynthetic metrics showed minimal fluctuation on day 5 of the salinity stress, while considerable change was seen on day 10 of the stress.

In mung bean varieties treated with Si 10 days after 20 mM NaCl/+Si treatment, net-photosynthetic rate was much higher by 70% than in the control group. This difference was especially notable in Co7(Gg), and Co8 varieties. Comparably, plants treated with Si alone showed a 30–40% increase in transpiration rate (**Figure 4**), whereas Co7(Gg) and Co8 varieties had a 20% increase under Si plus 20 mM NaCl treatment. **Figure 5** displays a substantial shift in stomatal conductance. All mung bean varieties exhibited greater stomatal conductance with Si treatment, and it was also noted that Si, in particular with Co7(Gg) and Co8, attenuated the impact at both 10 and 20 mM NaCl. In all mung bean varieties, the quantum yield of PSII was severely reduced in the latter stages of salt stress (10 days), but it dramatically increased following Si supplementation (**Figure 6**). Furthermore, in Co7(Gg) and Co8 variety mung bean, Si levels were considerably greater when given alone compared to control plants.

### Relative water content

**Figure 7:**
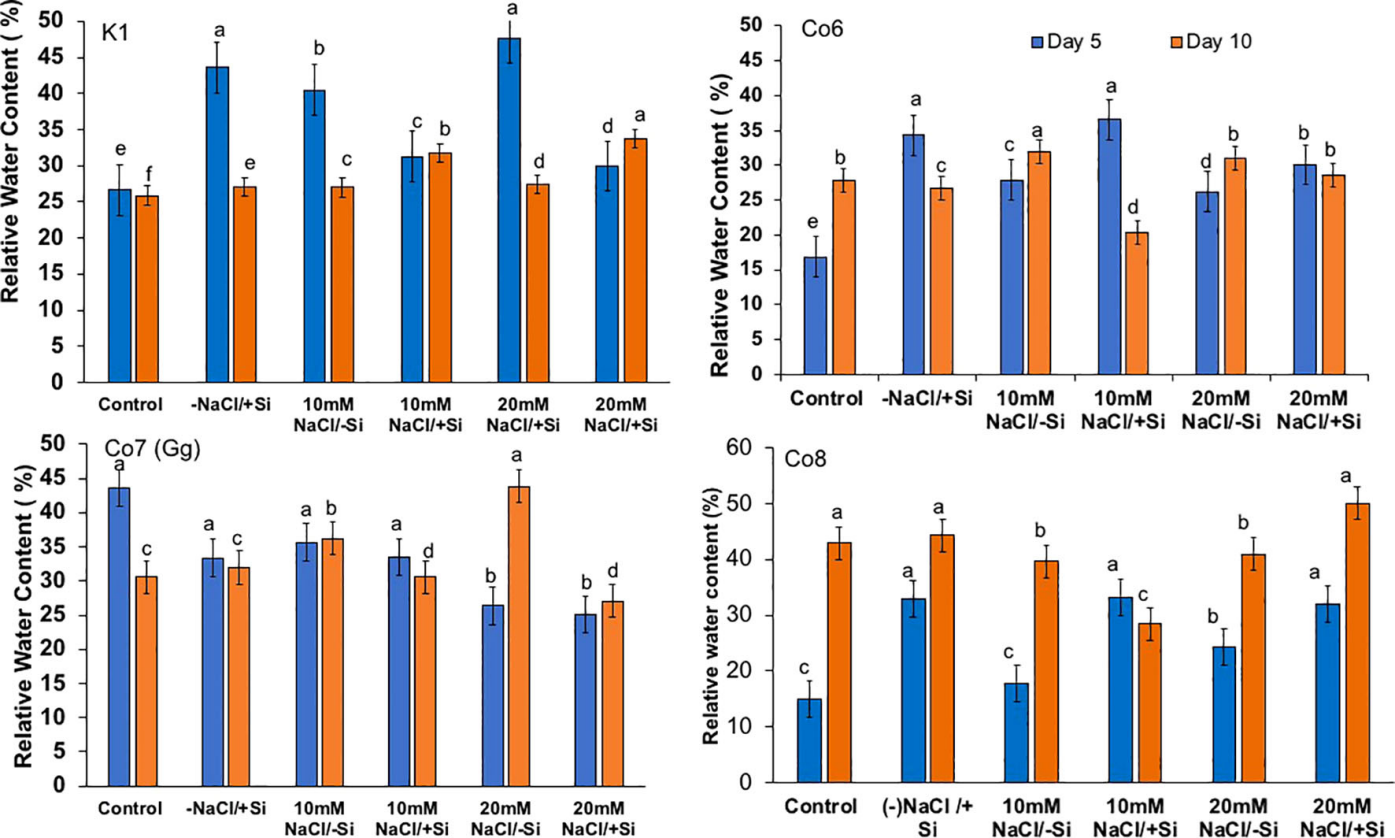
Changes in relative water content in mung bean varieties (*Vigna radiata L.*); K1, Co6, Co7(Gg), and Co8 under 5 mM Si supply and salinity stress after six treatments, (i) Control, (ii) -NaCl+Si (iii) 10 mM NaCl/-Si (iv) 10 mM NaCl/+ Si (v) 20 mM NaCl/–Si (vi) 20 mM NaCl/+Si for a period of 10 days. Vertical bars indicate Mean ± SE of the means for n = 4. Means denoted by the different letter are significantly different at P ≤ 0.05.

Plants’ water potential is a vital physiological mechanism for a number of metabolic activities, and it often alters during stress. As we were examining the impacts of salt stress in this work, we also examined the relative water potential of the leaves (**Figure 7**). When Si was supplied during salt stress, mung bean RW potential was recovered by 30%. All varieties had an influence on restoring RW, with Co7(Gg) and Co8 showing a somewhat stronger impact by more than 30%.

### Reactive oxygen species ROS/antioxidants

**Figure 8:**
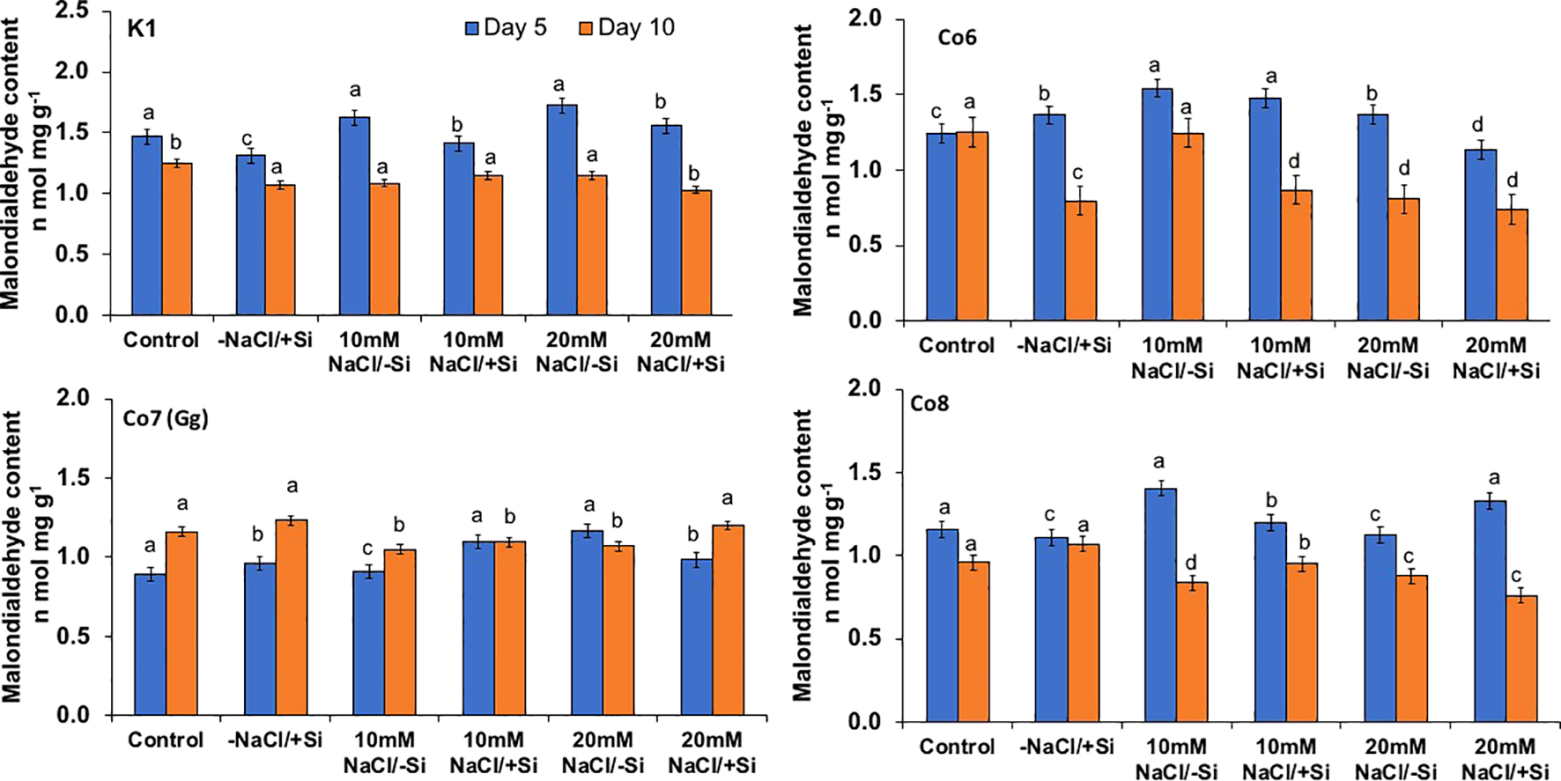
Changes in malonaldehyde content rate in mung bean varieties (*Vigna radiata L.*); K1, Co6, Co7(Gg), and Co8 under 5 mM Si supply and salinity stress after six treatments, (i) Control, (ii) -NaCl+Si (iii) 10 mM NaCl/-Si (iv) 10 mM NaCl/+ Si (v) 20 mM NaCl/–Si (vi) 20 mM NaCl/+Si for a period of 10 days. Vertical bars indicate Mean ± SE of the means for n = 4. Means denoted by the different letter are significantly different at P ≤ 0.05.

**Figure 9:**
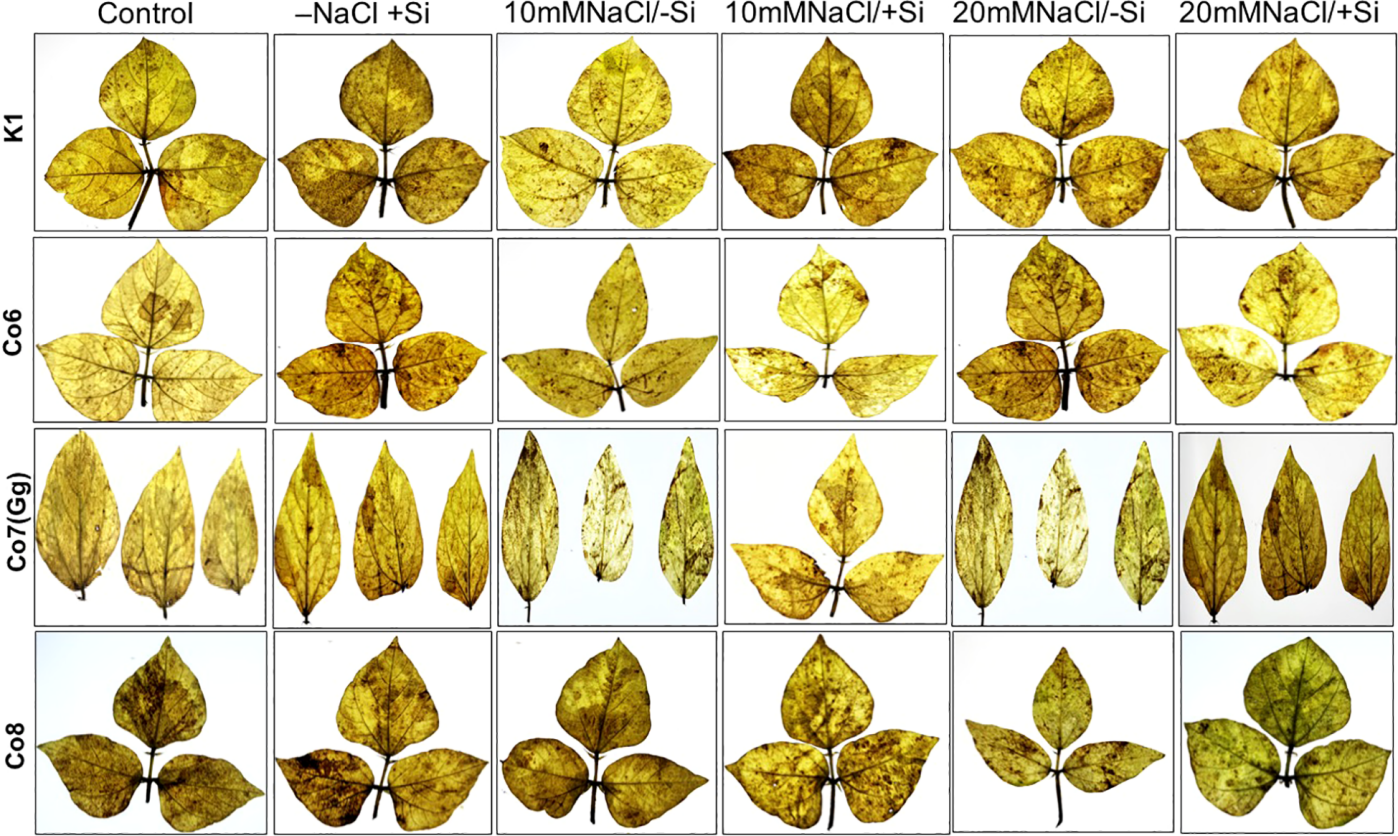
DAB staining for H_2_O_2_ localization in mung bean varieties (*Vigna radiata L.*); K1, Co6, Co7(Gg), and Co8 under 5 mM Si supply and salinity stress after six treatments, (i) Control, (ii) -NaCl+Si (iii) 10 mM NaCl/-Si (iv) 10 mM NaCl/+ Si (v) 20 mM NaCl/–Si (vi) 20 mM NaCl/+Si for a period of 10 days.

**Figure 10:**
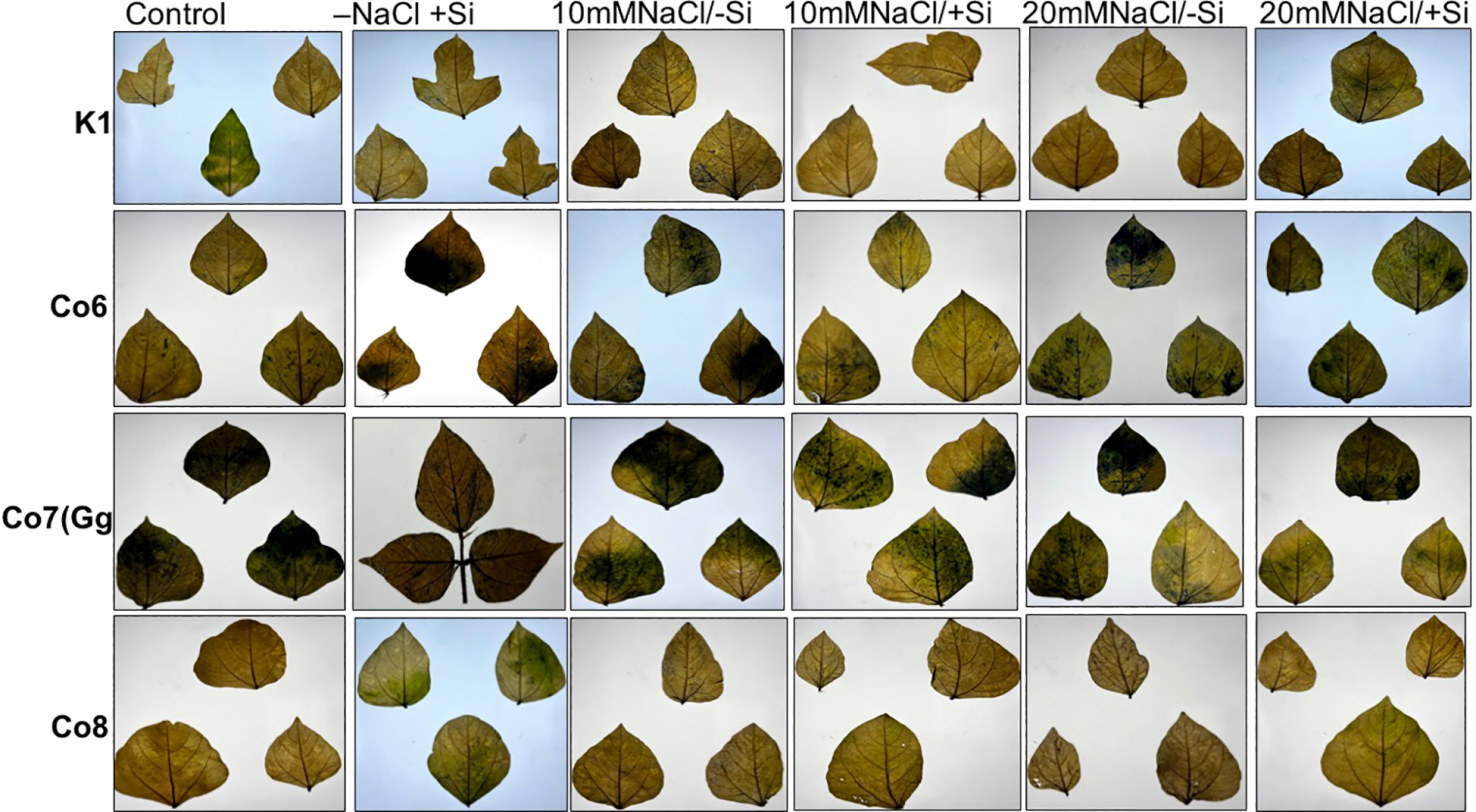
NBT staining for O_2–_^1^ localization in mung bean varieties (*Vigna radiata L.*); K1, Co6, Co7(Gg), and Co8 under 5 mM Si supply and salinity stress after six treatments, (i) Control, (ii) -NaCl+Si (iii) 10 mM NaCl/-Si (iv) 10 mM NaCl/+ Si (v) 20 mM NaCl/–Si (vi) 20 mM NaCl/+Si for a period of 10 days.

Salinity stress mostly manifests itself at the cellular level through the generation of free radicals and oxidative stress. Malonaldehyde content (MDA) and oxidative stress localizations were analyzed in order to investigate the oxidative damages (**Figures 8**–**10**). When exposed to salt stress, the MDA level of all mung bean cultivars rose significantly by 20%; however, this content was reversed when Si was provided (**Figure 8**). The results also made it clear that Co7(Gg) exhibited more mitigation than the other mung bean varieties. Under salt stress, all mung bean types showed increased levels of the isomer of oxidative stress, H_2_O_2_, which appeared as brownish spots or patches (**Figure 9**), but these levels decreased when Si was supplied. In a similar vein, NBT stanning revealed blue color spots in every variety of mung bean, which indicated the production of free radicals (**Figure 10**). The blue spots were more visible when under 20 mM NaCl/-Si treatments but lesser when Si was supplied.

**Figure 11:**
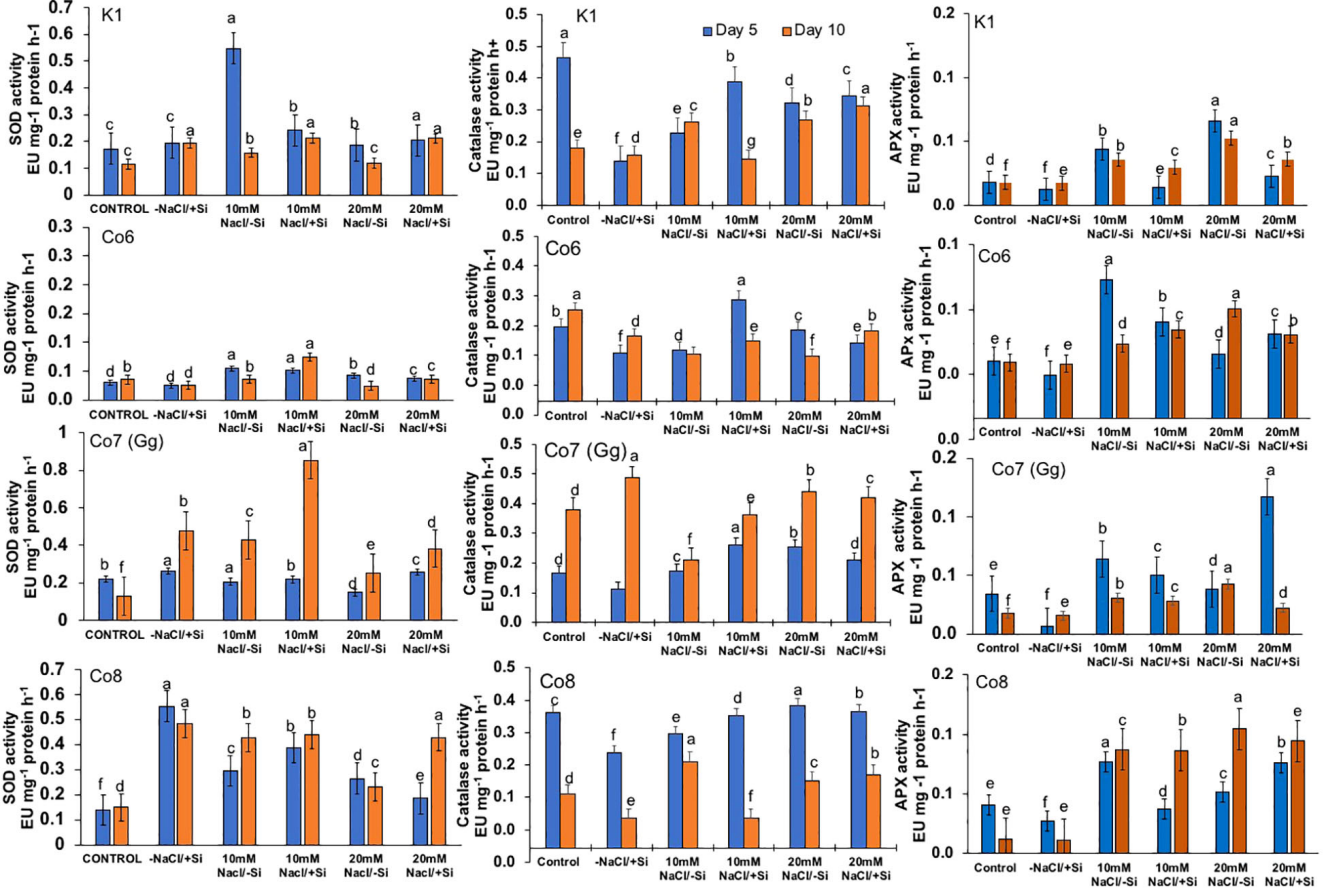
Changes in antioxidative enzymes superoxide dismutase, catalase, and ascorbate peroxidase in mung bean varieties (*Vigna radiata L.*); K1, Co6, Co7(Gg), and Co8 under 5 mM Si supply and salinity stress after six treatments, (i) Control, (ii) -NaCl+Si (iii) 10 mM NaCl/-Si (iv) 10 mM NaCl/+ Si (v) 20 mM NaCl/–Si (vi) 20 mM NaCl/+Si for a period of 10 days. Vertical bars indicate Mean ± SE of the means for n = 4. Means denoted by the different letter are significantly different at P ≤ 0.05.

**Figure 12:**
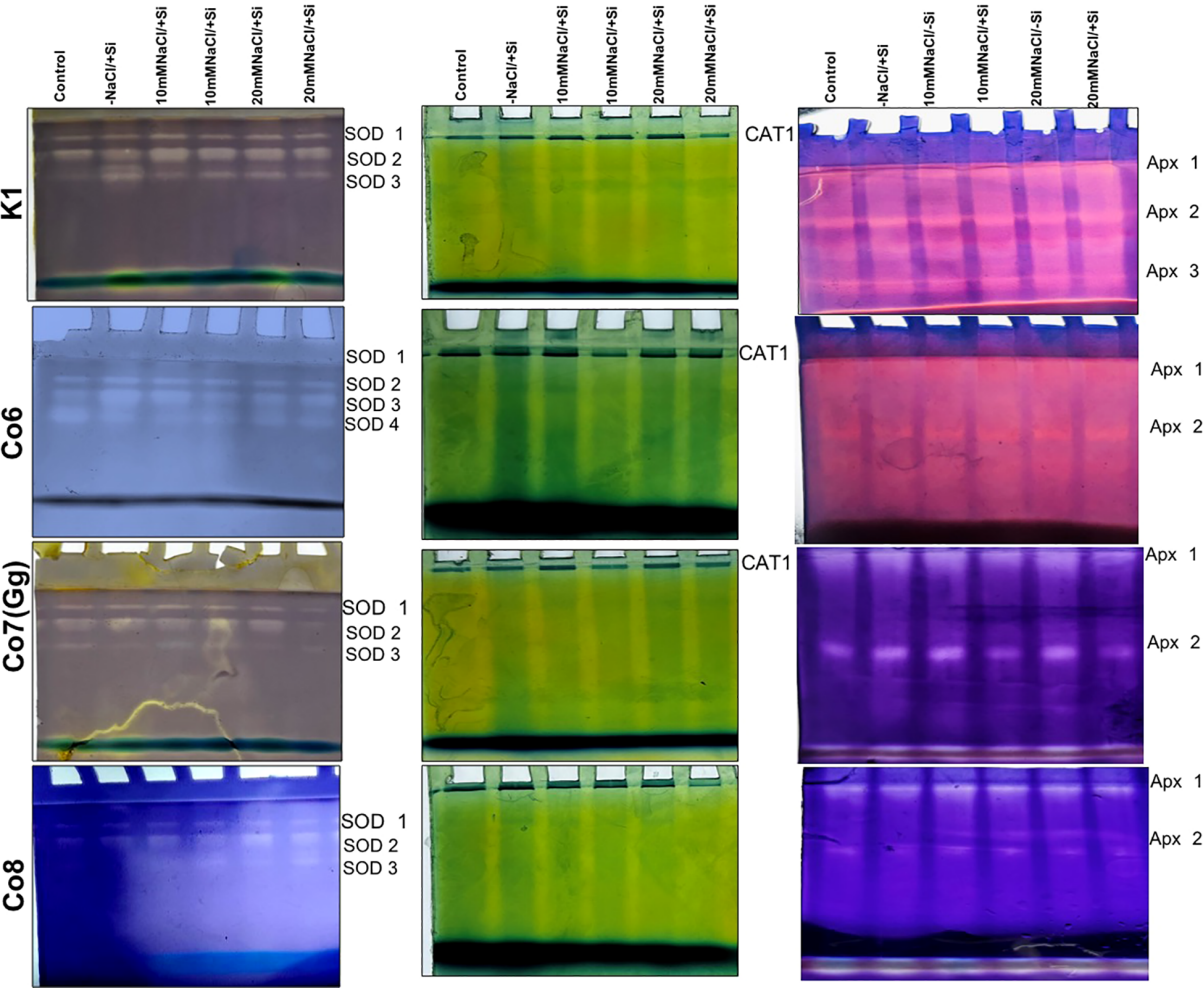
Changes in isozymes of antioxidative enzymes superoxide dismutase, catalase, and ascorbate peroxidase in mung bean varieties (*Vigna radiata L.*); K1, Co6, Co7(Gg), and Co8 under 5 mM Si supply and salinity stress after six treatments, (i) Control, (ii) -NaCl+Si (iii) 10 mM NaCl/-Si (iv) 10 mM NaCl/+ Si (v) 20 mM NaCl/–Si (vi) 20 mM NaCl/+Si for a period of 10 days.

Salinity stress had an impact on superoxide dismutase (SOD) activity, at 10 mM NaCl, SOD activity was substantially lower than that of the control and considerably highest at 20 mM NaCl. When compared to other varieties, the Co7(Gg) and Co8 showed higher SOD enzyme activity (**Figure 11**). The isozymes of SOD, namely the expression levels of SOD1-SOD4, were later shown to be concurrent with the activity of SOD (**Figure 12**). In mung bean varieties under salt stress, there was a noticeable increase in the expression levels of SOD isozymes, which was mitigated by the supply of silicon. In a similar pattern, catalase activity was considerably higher in response to increasing salt stress concentrations (**Figure 12**) and recovered in response to Si supply, with greater restoration shown in Co7(Gg) and Co8 cultivars. Despite the fact that only one isozyme expression level was seen, the results were consistent with our isozyme activities of catalase (**Figure 12**). Additionally, it was shown that when Si was supplied, ascorbate peroxidase activity (APX) was further lowered while being highly elevated under salt stress conditions (**Figure 12**). Under salt stress conditions, the isozyme expression level was quite high, and it simultaneously decreased with application of Si. All of the enzyme activity and their isozymes were generally more induced under salt stress conditions and reduced upon application of Si.

### Total soluble and multiprotein complex proteins

**Figure 13:**
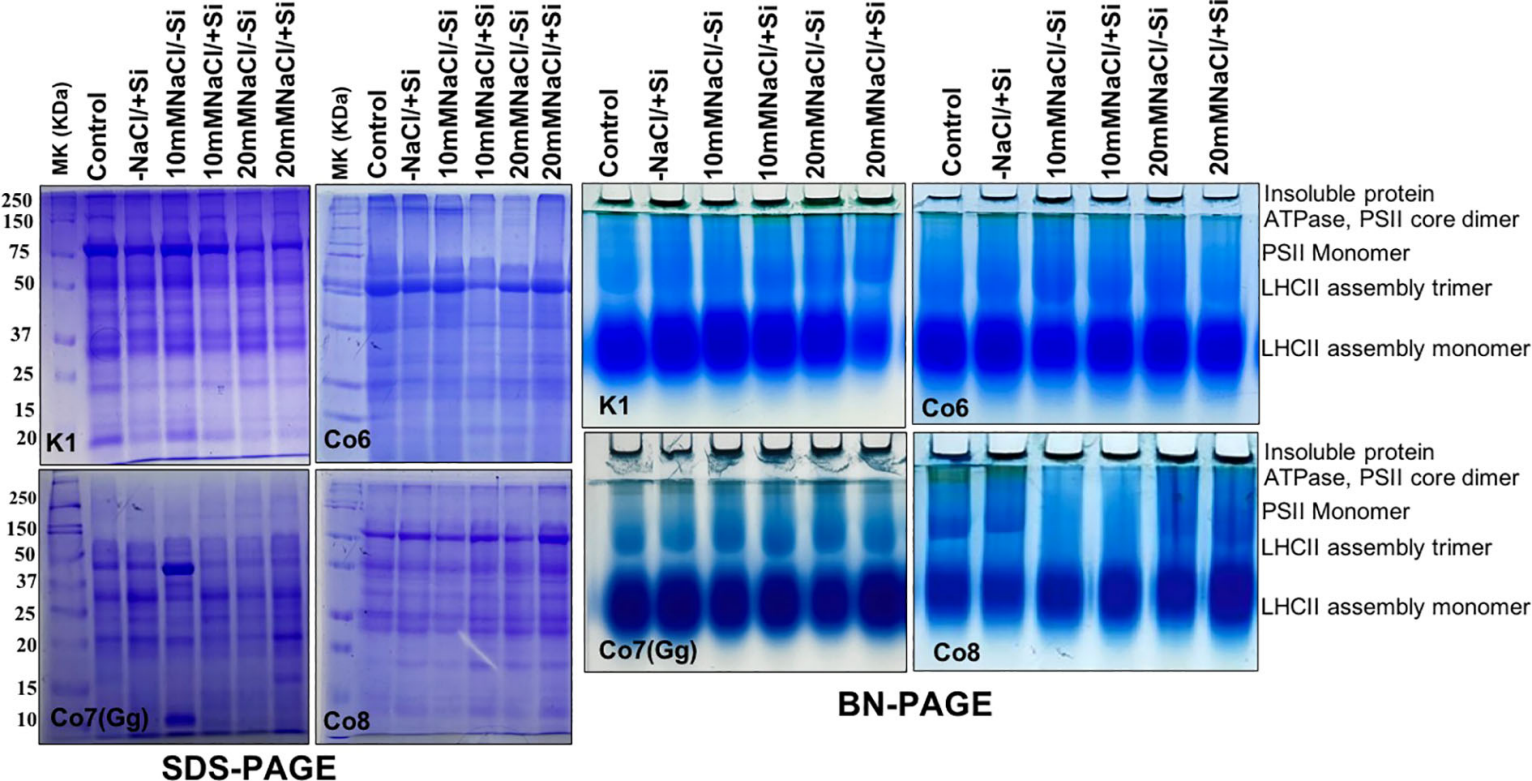
Protein profile (SDS-PAGE) and thylakoidal protein complexes of chloroplast (BN-PAGE) in mung bean varieties (*Vigna radiata L.*); K1, Co6, Co7(Gg), and Co8 under 5 mM Si supply and salinity stress after six treatments, (i) Control, (ii) -NaCl+Si (iii) 10 mM NaCl/-Si (iv) 10 mM NaCl/+ Si (v) 20 mM NaCl/–Si (vi) 20 mM NaCl/+Si for a period of 10 days.

Proteins are essential components of all plant metabolic activities. In all mung bean varieties, total protein expression levels were either up- or down-regulated in response to salt stress; however, the expression levels did not indicate any particular hypothesis. Whereas, quantitative analysis of total soluble proteins indicated reduction under salinity stress nevertheless, increased while Si was supplied (**Supplementary Figure 2**). In order to examine specific proteins, namely chloroplast/thylakoidal proteins, the blue native page showed how the expression level of certain proteins, including ATPase and PSII-monomer, was lowered in mung bean varieties under salt stress (**Figure 13**). Then, it was observed that plants given Si had higher levels of these proteins’ expression. The Co7(Gg) and Co8 varieties showed greater overall expression levels than the K1 and Co6 varieties.

### Yield observations

According to our pot culture trials, our field investigation was crucial in supporting our hypothesis that Si can assist the mung bean cultivars in reducing the salt stress (**Supplementary Figure 3**). Salinity-stressed mung bean varieties showed a substantial reduction in yield metrics, including length of plant, biomass, number of flowers, number of pods, number of seeds per 100 pods, and chlorophyll (SPAD), as compared to control varieties (**Table 1**, **Supplementary Data**). Nevertheless, under salinity conditions, the aforementioned yield characteristics improved when Si was supplied. In fact, when Si was given alone, mung bean varieties showed a relatively high number of pods (**Supplementary Figure 3**). According to the our studies, Si may function as fertilizer both with and without salt. In addition, the field data showed that Co7(Gg) and Co8 varieties have the potential to provide higher yields than K1 and Co6 varieties.

**Table 1:** Yield data in field conditions in mung bean varieties under salinity stress and Si supply.

Variety	Treatment	Plant height	Biomass	No of flowers	SPAD	No of Pods	No of seeds /100 pods
K1	-NaCl/-Si	44 ± 0.77^b^	40.9 ± 8.2^a^	6.7 ± 0.7^a^	54.73 ± 1.42^d^	6.33 ± 0.67^a^	7.4 ± 0.92^a^
	-NaCl/+Si	40.8 ± 0.37b^c^	63.0 ± 21.5^a^	2.7 ± 1.2^a^	52.83 ± 5.45^d^	6.00 ± 0.58^a^	9.4 ± 0.50^a^
	10mM NaCl /-Si	38.8 ± 1.39^c^	74.2 ± 18.5^a^	4.7 ± 1.8^a^	63.97 ± 2.03^a^	6.67 ± 1.45^a^	6 ± 1.04^a^
	10mM NaCl /+Si	36.4 ± 1.43^cd^	40.5 ± 6.00^a^	7.3 ± 1.3^a^	59.33 ± 2.95^b^	3.33 ± 0.88^a^	8.2 ± 0.8^a^
	20mM NaCl /-Si	33.6 ± 1.63^d^	25.4 ± 5.6^b^	10.0 ± 3.6^a^	56.23 ± 3.04^c^	5.00 ± 1.00^a^	8.8 ± 1.2^a^
	20mM NaCl /+Si	50.4 ± 0.51^a^	26.78 ± 14.0^b^	3.3 ± 0.3^a^	55.67 ± 3.56^d^	4.67 ± 1.20^a^	8 ± 1.14^a^
Co6	-NaCl/-Si	40.8 ± 0.84^ab^	13.8 ± 5.25^b^	1.33 ± 0.33^a^	55.33 ± 1.43^c^	7.6 ± 3.17^ab^	10.2 ± 0.8^a^
	-NaCl/+Si	34.4 ± 2.78^bc^	22.6 ± 9.02^bc^	12.67 ± 2.19^b^	57.57 ± 1.33^b^	11.6 ± 1.46^ab^	8.8 ± 0.86^ab^
	10mM NaCl /-Si	22.8 ± 0.73^d^	35.5 ± 3.56^a^	2.00 ± 0.58^a^	55.50 ± 1.46^c^	13.6 ± 0.92^a^	7 ± 0.60^ab^
	10mM NaCl /+Si	33.8 ± 1.46^c^	20.9 ± 8.41^b^	3.00 ± 2.00^a^	56.43 ± 3.09^c^	8.6 ± 2.11^ab^	8.5 ± 0.53^a^
	20mM NaCl /-Si	36.2 ± 0.58^bc^	16.4 ± 5.50	3.00 ± 1.00^a^	58.77 ± 1.01^b^	4.2 ± 1.06^a^	8.2 ± 0.8^ab^
	20mM NaCl /+Si	45.0 ± 1.67^a^	53.5 ± 10.18^a^	2.00 ± 1.00^a^	60.03 ± 1.55^1^	5 ± 0.83^b^	5 ± 1^b^
Co7(Gg)	-NaCl/-Si	41.4 ± 0.93^a^	53.7 ± 14.52^a^	2 ± 1.00^a^	41.37 ± 0.90^d^	8.33 ± 1.33^a^	10 ± 0.7^a^
	-NaCl/+Si	39.6 ± 0.40^a^	17.9 ± 2.95^c^	17.67 ± 3.18^a^	50.90 ± 1.44^c^	4.33 ± 0.33^ab^	7.6 ± 0.9^ab^
	10mM NaCl /-Si	34.6 ± 0.51^b^	21.9 ± 7.17^b^	11.33 ± 7.42^a^	55.93 ± 1.88^b^	6.00 ± 1.00^ab^	8 ± 0.9^ab^
	10mM NaCl /+Si	35 ± 1.00^b^	44.48 ± 13.8^b^	11 ± 4.18^a^	54.13 ± 2.57^a^	5.33 ± 0.88^ab^	5.4 ± 0.5^b^
	20mM NaCl /-Si	42.4 ± 0.81^a^	31.6 ± 4.73^b^	5.67 ± 2.33^a^	54.77 ± 2.05^a^	3.33 ± 0.88^b^	8.8 ± 0.7^a^
	20mM NaCl /+Si	34.6 ± 1.36^b^	32 ± 7.60^b^	10.00 ± 2.00^a^	52.73 ± 2.83^c^	5.67 ± 0.88^ab^	8.8 ± 0.6^a^
Co8	-NaCl/-Si	39.6 ± 0.51^a^	13.6 ± 30.6^a^	8.00 ± 1.53^bc^	54.33 ± 1.33^a^	6.33 ± 0.67^b^	9 ± 1.3^a^
	-NaCl/+Si	31.2 ± 0.73^b^	8.5 ± 3.38^a^	9.33 ± 1.45^abc^	55.40 ± 1.92^a^	6.00 ± 0.58^a^	6.20 ± 0.9^a^
	10mM NaCl /-Si	41.4 ± 1.36^a^	11.8 ± 4.18^a^	8.33 ± 2.03^abc^	54.47 ± 3.30^a^	6.67 ± 1.45^b^	8.6 ± 0.81^a^
	10mM NaCl /+Si	40.8 ± 0.37^a^	5.3 ± 0.86^a^	13.67 ± 3.18^ab^	53.77 ± 0.93^b^	3.33 ± 0.88^b^	10 ± 0.94^a^
	20mM NaCl /-Si	34.6 ± 1.60^b^	16.1 ± 3.69^a^	18.33 ± 2.85^a^	50.30 ± 2.63^b^	5.00 ± 1.00^ab^	8 ± 0.54^a^
	20mM NaCl /+Si	24.8 ± 0.73^c^	14 ± 3.78^a^	1.33 ± 0.33^c^	54.43 ± 1.88^a^	4.67 ± 1.20^b^	7.4 ± 1.2^a^

## Discussion

Abiotic stressors, particularly salinity in soil and water, significantly impede agricultural yield worldwide (Shrivastava and Kumar, 2015). Reports indicate that 8–10% of irrigated land, equivalent to one-third of food-producing land, is affected by salinity (Munns and Tester, 2008; FAO, 2021; Parida and Das, 2005; Wang et al., 2003; Shahid et al., 2018). Furthermore, it is projected that, by the middle of the twenty-first century, salinity will impact 50% of all agricultural land (Wang et al., 2003). The area affected by salinity is expanding at a rate of 10% per year due to several factors, including limited precipitation, the weathering of native rocks, high evaporation rates, poor agricultural practices, and irrigation with saline water (Parida and Das, 2005). Compounding this issue are persistent patterns of global warming and climate change. Therefore, improving agricultural plant resistance to abiotic stressors is crucial for reversing the decline in food production and meeting the growing global demand for food (Shahid et al., 2018). A significant 50% increase in grain yields of major crops such as wheat, rice, and maize is necessary to establish a sustainable food supply (Parida and Das, 2005; FAO, 2021; Munns and Tester, 2008).

Addressing these challenges requires a deep understanding of plant responses at the cellular level and the exploration of strategies to alleviate salt stress in soil. Among various agricultural practices, the use of beneficial nutrients has gained attention. Silicon (Si), classified as a quasi-essential element, shares striking similarities with carbon; however, its specific role in plant life remains less defined. Silicon is recognized as a central element in various fields, including artificial intelligence (AI) and nanobiotechnology, and can act as an informational molecule akin to nucleic acids. Additionally, silicon’s potential to bond with different chemical species resembles carbon, allowing it to serve as a structural candidate, such as proteins. In India, legumes, essential staple food crops, are consumed more than they are cultivated. These crops are often grown in alkaline soils or irrigated with water of higher electrical conductivity. The current study focuses on examining the response of mung bean cultivars to salinity stress and the potential role of silicon in ameliorating its effects.

Salinity stress adversely impacts plant morphology, physiology, and biochemistry (Parida and Das, 2005; Wang et al., 2003; Shahid et al., 2018). Excessive salinity reduces plant growth, biomass, yield, photosynthesis, and water use efficiency (Shahid et al., 2018). It also leads to ion toxicity and physiological changes, including alterations in photosynthesis, transpiration, and stomatal conductance, which ultimately result in decreased crop yield. Various adaptation and mitigation strategies have been implemented to combat the negative effects of elevated soil salinity (Ashraf, 2004). While these strategies may help ensure sustainable food production, managing salinity stress is particularly challenging due to its multigenic and quantitative nature (Munns and Tester, 2008).

Our findings show significant reductions in plant height, biomass, flower counts, pod counts, and seed production among mung bean varieties (**Table 1**, **Supplementary Data**), accompanied by decreased photosynthetic parameters such as net photosynthesis, transpiration rate, stomatal conductance, and PSII quantum yield (**Figures 3**-**6**). In contrast, the application of silicon notably restored these measurements, particularly in the Co7(Gg) and Co8 varieties, while K1 and Co6 showed less improvement. The results suggest that silicon significantly enhances the morphology (**Figure 1**), yield, and photosynthetic physiology of mung bean varieties Co7(Gg) and Co8.

Silicon promotes suberization, lignification, and silicification, reinforcing cell walls and providing mechanical support for monocots, pteridophytes, and some dicots (Ma et al., 2001). The binding of Si with cell-wall hemicellulose has been associated with improved structural stability (Coskun et al., 2019), which is particularly beneficial under water deprivation conditions. Silica deposition results (**Figure 2**) demonstrate that bio-silicification—characterized by the polymerization of silicic acid within the apoplast—creates an amorphous silica barrier that mitigates both biotic and abiotic stresses (Debona et al., 2017; Epstein, 1999).

Under salt stress, cells are at risk due to the overproduction of reactive oxygen species (ROS), which can lead to lipid peroxidation, protein oxidation, nucleic acid damage, enzyme inhibition, and activation of pathways that result in programmed cell death (Mittler, 2002; Gill and Tuteja, 2010). Non-enzymatic antioxidant molecules such as ascorbate, alkaloids, flavonoids, phenolic compounds, proline, glutathione, α-tocopherol, and carotenoids are activated to scavenge ROS. Antioxidant enzymes involved in the ascorbate-glutathione (AsA-GSH) cycle include GR, MDHAR, APX, and DHAR. Several studies have documented increased activity of the plant’s antioxidant defense system in response to salinity-induced oxidative stress (Gong et al., 2005; Gong et al., 2010; Zhu and Gong, 2019; Debona et al., 2017; Coskun et al., 2019; Epstein, 1999; Gill and Tuteja, 2010). In this context, silicon is reported to alleviate the adverse effects of salinity by strengthening the antioxidant defense capabilities of crops.

The present study demonstrates that salinity stress leads to the formation of ROS, indicated by stress markers such as MDA content, H_2_O_2_, and O_2_^-^ (**Figures 8**-**10**) in mung bean varieties. However, silicon application significantly mitigates these stress markers by increasing the activities of antioxidative enzymes, including SOD, APX, CAT, and their isozymes. Thus, our findings suggest that silicon supplementation can reduce the adverse effects of salinity by regulating the antioxidant defense system, which, in turn, decreases lipid peroxidation, maintains membrane integrity, and reduces plasma membrane permeability. Notably, Si-treated and non-Si-treated mung bean varieties exhibit different responses under salinity stress, highlighting the protective role of silicon against salinity. Furthermore, silicon enhances crops’ ability to defend against free radicals, mitigating the negative impacts of salt. Our research revealed that the application of silicon restored the negative effects on mung bean varieties by reducing ROS formation in the form of MDA content, H_2_O_2_, and O_2_^-^ (**Figures 8**-**11**) and increasing antioxidative enzyme activities and their isozymes (**Figure 12**).

Our study demonstrates that salinity stress significantly reduced morphological traits (plant height, biomass, flower and pod counts, seed yield) and photosynthetic parameters (net photosynthesis, stomatal conductance, transpiration rate, and PSII quantum yield) across mung bean varieties (**Figures 3**-**6**; **Table 1**). However, silicon supplementation mitigated these negative effects, particularly in Co7(Gg) and Co8 cultivars, which showed greater improvements in growth, yield, and photosynthetic efficiency compared to K1 and Co6. The superior performance of Co7(Gg) and Co8 may be attributed to several factors: Enhanced Silicon Uptake and Deposition – Co7(Gg) and Co8 exhibited higher silicon accumulation in cell walls and leaf tissues, which reinforced structural stability and reduced cellular damage under osmotic and ionic stress. Stronger Antioxidant Defense – These cultivars displayed higher basal and silicon-induced activities of key antioxidative enzymes (SOD, APX, CAT) and their isozymes, leading to more efficient scavenging of ROS and lower oxidative stress markers (MDA, H_2_O_2_, O_2_^-^) (**Figures 8**-**10**). Better Photosynthetic Regulation – The maintenance of stomatal conductance, PSII efficiency, and net photosynthesis in Co7(Gg) and Co8 indicates an improved capacity to sustain carbon assimilation under salinity, likely due to silicon-mediated protection of chloroplast membranes and photosynthetic proteins. Genotype-Specific Stress Tolerance Traits – Inherent differences in genetic makeup, such as salt exclusion, osmotic adjustment, or enhanced ROS signaling and antioxidant responsiveness, may underpin the differential responses observed between cultivars.

Silicon’s multifaceted role in enhancing cell-wall integrity, modulating antioxidant defenses, and maintaining photosynthetic efficiency collectively mitigates the adverse effects of salinity in mung bean. Our findings show that silicon-treated Co7(Gg) and Co8 cultivars experienced reduced ROS formation, lower lipid peroxidation, preserved membrane integrity, and improved growth and yield. These effects highlight silicon’s potential as a practical agronomic intervention to improve legume resilience in saline soils. Notably, varietal differences underscore the importance of selecting genotypes with inherently higher silicon responsiveness for optimal stress mitigation.

Overall, the results indicate that silicon supplementation can lessen the negative effects of salt by modulating the antioxidant defense system. This modulation may reduce lipid peroxidation, preserve membrane integrity, and minimize plasma membrane permeability. The findings suggest that silicon enhances antioxidant activity, helping plants withstand salinity stress, and that there are distinct responses to salt stress between treated and untreated plants. It should be noted that the majority of these findings derived from pot culture align with our field experiments. In conclusion, silicon supplementation represents an effective strategy to counteract salinity-induced oxidative stress and structural damage in mung bean. Co7(Gg) and Co8, due to their superior silicon uptake, antioxidant capacity, and photosynthetic resilience, demonstrated better performance under stress, suggesting that genotype-specific management combined with silicon application can enhance crop productivity in saline-prone regions. These insights bridge pot-based mechanistic studies with field applicability, providing a comprehensive framework for improving legume tolerance to salinity.

## Conclusions

Sustainable and resilient food production is a key component for future food security for world population projected to be more than ten billion by 2050. Due to climate change irrigated water and cultivable land has become high in salinity stress that leads to reduction in crop productivity. Legumes being one of the major diet worldwide for protein consumption are unfortunately grown in calcareous and alkaline soil conditions having higher electrical conductivity thus has reduced its productivity in the last few years. To achieve higher crop productivity several agricultural practices or biotechnological approached are being practiced such as use speed breeding, use of protected structures, recombinant DNA technologies etc. but for crops like legumes more farmer friendly and economically acceptable technology is required. In this perspective we have highlighted an easy and a farmer friendly technology i.e., Si fertigation application to reduce the effects of salinity stress both at pot and field levels in mung bean-one of the major leguminous crop. Silicon (Si) fertigation has emerged as a practical and effective strategy to mitigate salinity stress in mung bean varieties Co7(Gg) and Co8, leading to significant improvements in both physiological performance and yield. Under saline conditions, Si application enhanced shoot and root biomass, chlorophyll content, and photosynthetic efficiency, thereby boosting overall plant productivity. Crucially, Si supplementation bolstered the activity of key antioxidant enzymes—superoxide dismutase (SOD), catalase (CAT), and ascorbate peroxidase (APX)—which played a pivotal role in reducing oxidative stress by lowering hydrogen peroxide (H_2_O_2_) and superoxide (O_2_^-^) levels. This enzymatic enhancement contributed to improved membrane stability and nutrient uptake, further supporting plant health under saline stress. Additionally, Si supplementation modulated the expression of multiprotein complex proteins, suggesting a molecular basis for its role in salinity tolerance. From a practical applications, Si fertigation offers a cost-effective, farmer-friendly approach to enhancing mung bean productivity in saline-prone areas, aligning with sustainable agricultural practices and contributing to food security in the face of climate challenges.

## Data Availability

The original contributions presented in the study are publicly available. This data can be found here: https://zenodo.org/records/17718845.
