# Supplementary material for: Silicon fertigation alleviates salinity stress by enhancing morpho-physiological, photosynthetic, antioxidative responses, and yield in mung bean (Vigna radiata L.) varieties Co7(Gg) and Co8 under pot and field conditions
Source: Front Plant Sci. 2025 Dec 2;16:1693710. doi: 10.3389/fpls.2025.1693710 (PMC12705399; doi:10.3389/fpls.2025.1693710)
Supplement: Supplementary file 1 [file DataSheet1.pdf]

<u>FIELD LAYOUT</u>

Area: Mini Orchard **N**

Mung Bean (*Vigna radiata* L.)

Design: FRBD Replication: 3

Single Plot area: 4 x 3 sq meters total

No: of: Plots: 72

total area covered: 1392 Sq mts

Space between plots: 1 m

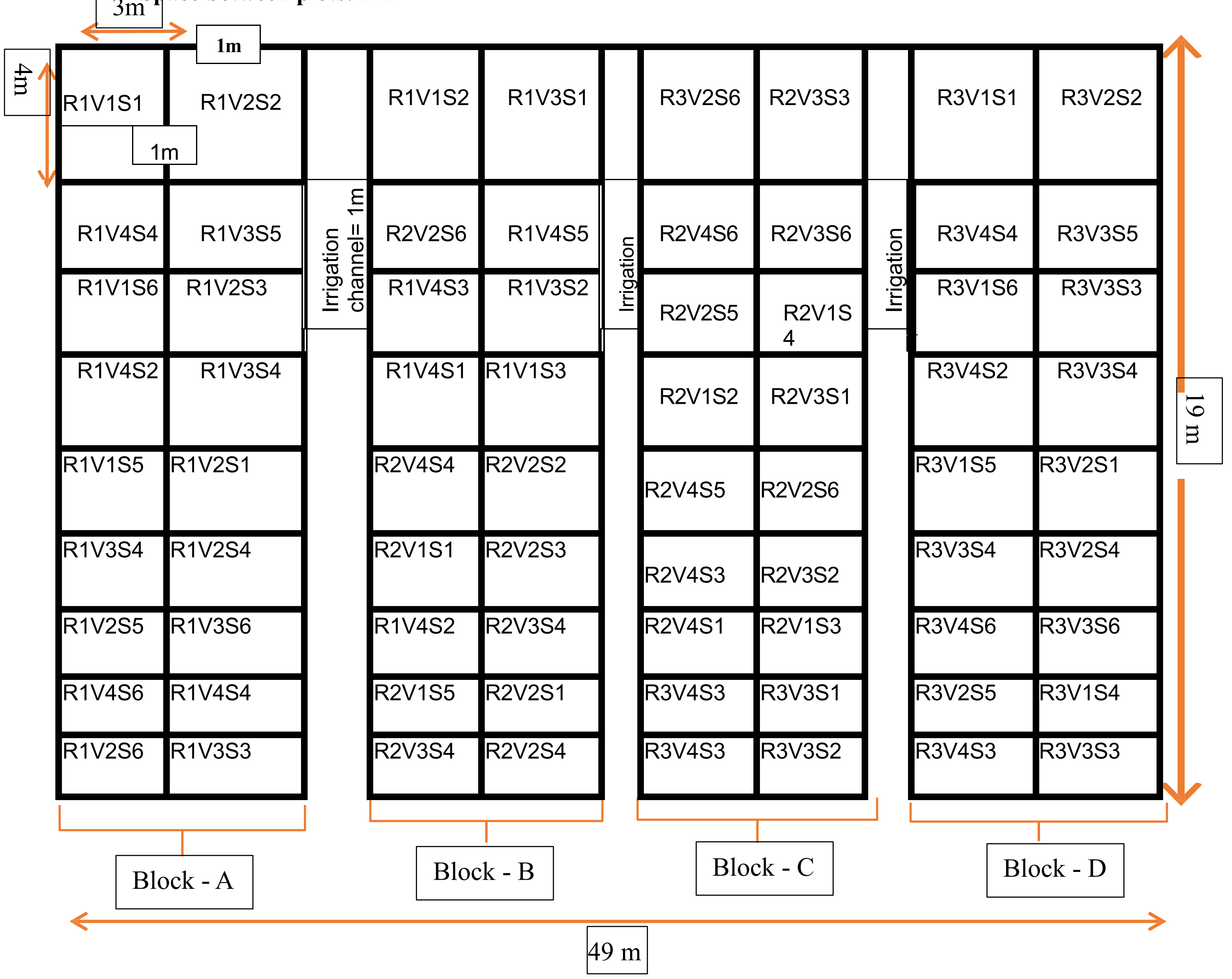

V1- K1; V2-Co - 6; V3 – Co 7 (Gg) and V4 – Co 8

S1-NaCl-Si; S2 -NaCl+Si; S3-10 mM NaCl/-Si; S4-10 mM NaCl/+ Si; S5-20 mM NaCl/–Si; and S6-20 mM NaCl/+Si
