# Supplementary material for: Silicon fertigation alleviates salinity stress by enhancing morpho-physiological, photosynthetic, antioxidative responses, and yield in mung bean (Vigna radiata L.) varieties Co7(Gg) and Co8 under pot and field conditions
Source: Front Plant Sci. 2025 Dec 2;16:1693710. doi: 10.3389/fpls.2025.1693710 (PMC12705399; doi:10.3389/fpls.2025.1693710)
Supplement: Supplementary file 2 [file DataSheet2.pdf]

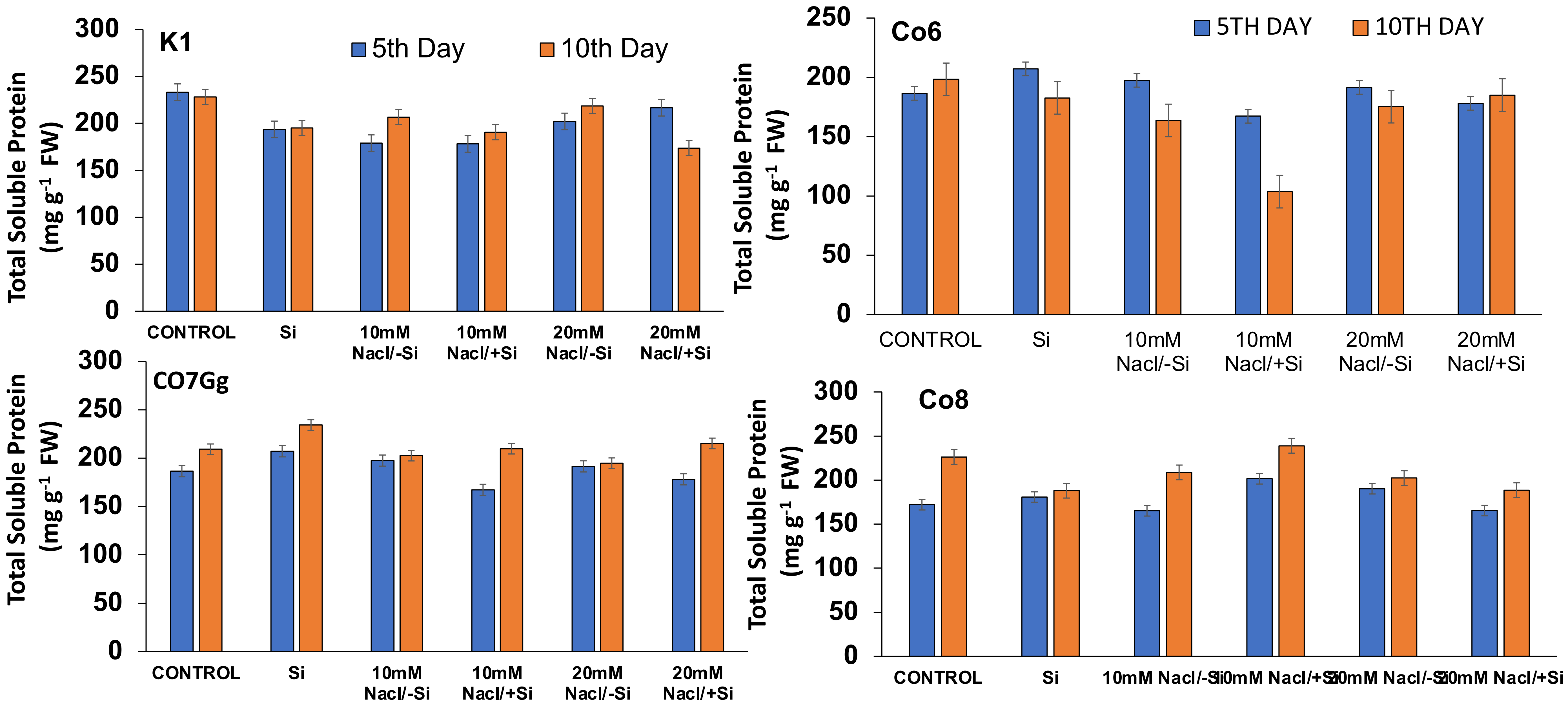

Changes in total protein content in mung bean varieties (*Vigna radiata L.*); K1, Co6, Co7Gg, and Co8 under 5 mM Si supply and salinity stress after six treatments, (i) Control, (ii) -NaCl+Si (iii) 10 mM NaCl/-Si (iv) 10 mM NaCl/+ Si (v) 20 mM NaCl/–Si (vi) 20 mM NaCl/+Si for a period of 10 days. Vertical bars indicate Mean±SE of the means for n = 4.
