# Supplementary figures and images for: Silicon fertigation alleviates salinity stress by enhancing morpho-physiological, photosynthetic, antioxidative responses, and yield in mung bean (Vigna radiata L.) varieties Co7(Gg) and Co8 under pot and field conditions
Source: Front Plant Sci. 2025 Dec 2;16:1693710. doi: 10.3389/fpls.2025.1693710 (PMC12705399; doi:10.3389/fpls.2025.1693710)

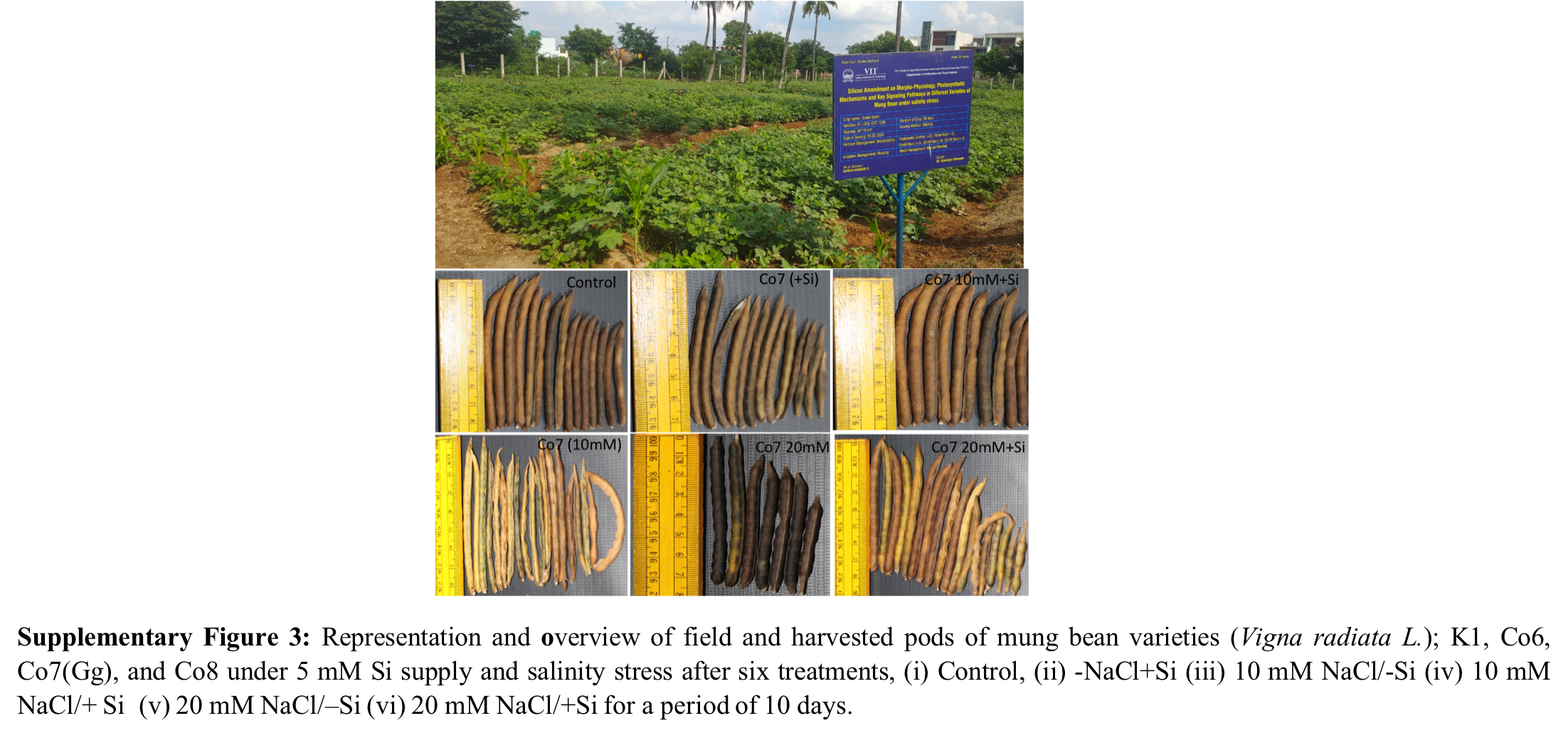

Supplement: Supplementary file 3 [file Image1.png]
