# Supplementary material for: Silicon fertigation alleviates salinity stress by enhancing morpho-physiological, photosynthetic, antioxidative responses, and yield in mung bean (Vigna radiata L.) varieties Co7(Gg) and Co8 under pot and field conditions
Source: Front Plant Sci. 2025 Dec 2;16:1693710. doi: 10.3389/fpls.2025.1693710 (PMC12705399; doi:10.3389/fpls.2025.1693710)
Supplement: Supplementary file 4 [file Table1.docx]

| **=** | | | **Weekly Weather Data – 2024** | | | | | | | | | |  | | | |
| --- | --- | --- | --- | --- | --- | --- | --- | --- | --- | --- | --- | --- | --- | --- | --- | --- |
|  | | | | | | | | | | | | | | | | |
| **S.No** | **Max** | **Min** | **Wet** | **Dry** | **RH 07.00am & 14.00pm** | | **Evop** | **Rain** | **W-S** | **W-D** | **Sun** | **Soil Temperature** | | | | |
|  |  |  |  |  |  |  |  |  |  |  |  | **5cm** | **10cm** | **15cm** | **20cm** | **30cm** |
| **1** | 29.0 | 20.5 | 24.0 | 24.5 | 88 | 58 | 3.6 | 0 | 5.16 | N-E | 5.10 | 23.4 | 24.0 | 24.5 | 25.2 | 26.2 |
| **2** | 29.0 | 20.0 | 24.0 | 24.5 | 88 | 54 | 4.7 | 0 | 4.65 | N-W | 6.10 | 22.8 | 23.5 | 24.1 | 24.9 | 26.1 |
| **3** | 29.5 | 20.0 | 24.0 | 24.5 | 87 | 57 | 3.7 | 0 | 5.42 | N-W | 6.30 | 22.7 | 23.2 | 23.8 | 24.7 | 25.8 |
| **4** | 29.0 | 19.0 | 24.5 | 24.0 | 89 | 52 | 3.9 | 0 | 5.05 | S-W | 7.00 | 23.7 | 24.5 | 25.2 | 26.0 | 27.1 |
| **5** | 31.0 | 21.0 | 23.5 | 24.5 | 89 | 49 | 4.1 | 0 | 5.11 | N-W | 6.00 | 23.8 | 24.7 | 25.7 | 26.2 | 27.2 |
| **6** | 32.0 | 23.0 | 20.0 | 21.0 | 85 | 41 | 5.5 | 0 | 4.58 | N-E | 7.40 | 24.7 | 25.5 | 26.8 | 28.1 | 29.8 |
| **7** | 32.0 | 22.0 | 20.5 | 22.0 | 86 | 40 | 5.5 | 0 | 5.71 | N-E | 7.40 | 25.7 | 26.4 | 27.5 | 28.5 | 30.4 |
| **8** | 33.0 | 23.0 | 22.0 | 23.0 | 88 | 42 | 4.7 | 0 | 4.85 | N-E | 7.00 | 26.1 | 26.5 | 27.7 | 28.2 | 30.5 |
| **9** | 33.0 | 23.0 | 21.0 | 22.0 | 87 | 42 | 5.08 | 0 | 5.97 | N-E | 8.10 | 24.9 | 26.0 | 27.4 | 28.7 | 31.1 |
| **10** | 36.0 | 20.0 | 21.0 | 22.0 | 84 | 43 | 4.2 | 0 | 6.31 | N-E | 8.50 | 25.0 | 26.1 | 27.8 | 28.8 | 30.4 |
| **11** | 36.5 | 22.0 | 23.5 | 25.0 | 89 | 46 | 4.5 | 0 | 6.46 | N-E | 8.15 | 27.5 | 28.7 | 30.0 | 31.4 | 32.7 |
| **12** | 36.0 | 22.5 | 23.5 | 25.0 | 88 | 46 | 4.2 | 0 | 6.25 | N-E | 9.00 | 28.0 | 28.8 | 30.2 | 31.8 | 33.2 |
| **13** | 37.0 | 22.5 | 23.5 | 26.0 | 88 | 42 | 4.07 | 0 | 6.22 | N-E | 9.10 | 26.0 | 29.0 | 31.0 | 32.0 | 34.0 |
| **14** | 38.0 | 24.0 | 23.5 | 25.5 | 85 | 43 | 5.38 | 0 | 6.18 | S-E | 9.15 | 27.5 | 28.8 | 30.1 | 31.3 | 33.5 |
| **15** | 29.0 | 24.5 | 24.0 | 25.5 | 89 | 46 | 5.2 | 0 | 6.42 | S-E | 8.30 | 28.2 | 28.8 | 30.2 | 31.4 | 33.4 |
| **16** | 39.0 | 24.5 | 24.0 | 25.5 | 84 | 41 | 5.1 | 0 | 6.82 | N-E | 9.30 | 28.1 | 29.2 | 30.7 | 32.4 | 33.8 |
| **17** | 39.0 | 23.0 | 24.0 | 26.0 | 85 | 43 | 4.5 | 0 | 5.94 | S-E | 9.00 | 29.1 | 30.2 | 31.1 | 32.8 | 34.5 |
| **18** | 40.0 | 24.0 | 24.5 | 25.5 | 85 | 40 | 5.8 | 0 | 6.04 | N-E | 9.15 | 28.2 | 28.8 | 29.8 | 31.4 | 33.4 |
| **19** | 39.0 | 25.0 | 23.9 | 25.5 | 86 | 42 | 5.1 | 0 | 6.78 | N-E | 8.45 | 29.7 | 30.1 | 31.0 | 32.2 | 33.7 |
| **20** | 38.0 | 26.5 | 24.5 | 26.0 | 87 | 43 | 4.8 | 0 | 7.10 | N-E | 6.75 | 27.7 | 28.0 | 29.0 | 30.7 | 33.5 |
| **21** | 38.0 | 26.5 | 24.5 | 26.0 | 87 | 43 | 4.8 | 0 | 7.10 | N-E | 7.15 | 27.4 | 27.9 | 28.5 | 29.4 | 32.0 |
| **22** | 39.5 | 26.5 | 24.0 | 25.5 | 87 | 41 | 4.3 | 37.0 | 7.26 | N-E | 8.00 | 28.4 | 28.7 | 29.8 | 31.0 | 31.5 |
| **23** | 36.5 | 24.5 | 25.0 | 26.5 | 87 | 49 | 3.5 | 43.5 | 7.20 | N-E | 6.30 | 27.7 | 28.2 | 28.8 | 29.8 | 31.8 |
| **24** | 37.0 | 26.0 | 24.5 | 25.5 | 87 | 48 | 4.3 | 35.0 | 6.30 | N-E | 7.10 | 29.1 | 30.2 | 30.7 | 32.0 | 33.2 |
| **25** | 37.5 | 25.5 | 24.0 | 25.5 | 86 | 47 | 4.1 | 32.9 | 6.21 | N-E | 8.00 | 27.2 | 27.7 | 28.4 | 29.8 | 31.2 |
| **26** | 37.0 | 25.5 | 25.0 | 27.0 | 84 | 47 | 5 | 0 | 5.51 | N-E | 7.00 | 26.8 | 27.1 | 28.2 | 30.1 | 32.1 |
| **27** | 36.0 | 26.0 | 23.5 | 26.0 | 80 | 51 | 4.3 | 70.0 | 5.41 | N-E | 7.20 | 26.8 | 27.4 | 28.2 | 29.0 | 30.2 |
| **28** | 36.0 | 26.0 | 24.0 | 26.5 | 81 | 50 | 4.9 | 164.0 | 6.48 | S-E | 7.50 | 26.8 | 26.8 | 28.0 | 28.8 | 30.0 |
| **29** | 34.0 | 25.0 | 23.5 | 26.5 | 79 | 47 | 4.0 | 27.0 | 5.73 | N-E | 6.00 | 26.5 | 26.7 | 27.1 | 28.1 | 29.2 |
| **30** | 35.0 | 26.0 | 25.0 | 27.0 | 82 | 46 | 5.2 | 0 | 6.27 | N-E | 6.30 | 27.0 | 27.1 | 28.4 | 28.7 | 29.4 |
| **31** | 36.0 | 24.5 | 24.5 | 27.0 | 80 | 47 | 4.8 | 0 | 5.23 | N-E | 7.00 | 27.8 | 28.0 | 28.5 | 29.5 | 31.2 |
| **32** | 35.0 | 26.5 | 24.5 | 26.0 | 82 | 47 | 3.8 | 103.0 | 5.04 | N-E | 7.00 | 27.2 | 27.2 | 28.4 | 29.0 | 30.5 |
| **33** | 35.0 | 26.5 | 25.0 | 27.0 | 83 | 46 | 4.0 | 5.0 | 4.94 | N-E | 8.00 | 27.7 | 27.7 | 28.5 | 29.2 | 30.7 |
| **34** | 36.0 | 26.5 | 25.7 | 27.5 | 82 | 47 | 4.0 | 30.0 | 4.38 | N-E | 8.40 | 29.7 | 29.8 | 30.5 | 31.8 | 32.5 |
| **35** | 35.3 | 25.5 | 24.5 | 26.5 | 83 | 52 | 3.9 | 42.0 | 5.94 | N-E | 8.15 | 26.7 | 27.0 | 27.7 | 28.8 | 30.1 |
| **36** | 35.0 | 24.0 | 24.5 | 26.5 | 80 | 56 | 2.5 | 187.0 | 5.06 | N-E | 5.30 | 27.0 | 27.1 | 27.8 | 28.7 | 29.7 |
| **37** | 34.5 | 25.0 | 24.5 | 26.5 | 83 | 50 | 3.7 | 0 | 6.43 | N-E | 6.20 | 27.4 | 27.4 | 27.8 | 29.1 | 30.5 |
| **38** | 34.5 | 24.0 | 24.0 | 26.5 | 83 | 48 | 3.8 | 42.0 | 7.34 | N-E | 6.40 | 27.1 | 27.2 | 27.8 | 28.7 | 29.7 |
| **39** | 35.5 | 26.0 | 23.5 | 26.0 | 81 | 54 | 4.3 | 75.0 | 5.53 | N-E | 7.00 | 26.7 | 27.2 | 28.0 | 28.5 | 29.8 |
| **40** | 35.5 | 22.5 | 22.0 | 24.0 | 86 | 47 | 4.5 | 63.0 | 5.85 | N-E | 7.00 | 25.0 | 25.8 | 27.2 | 28.1 | 29.7 |
| **41** | 35.0 | 22.0 | 23.0 | 24.5 | 87 | 46 | 4.4 | 70.0 | 4.98 | N-E | 7.30 | 25.4 | 26.1 | 26.7 | 27.8 | 29.5 |
| **42** | 35.5 | 22.5 | 23.5 | 25.5 | 83 | 49 | 4.1 | 48.0 | 5.27 | S-E | 7.00 | 26.7 | 27.1 | 28.2 | 29.1 | 30.1 |
| **43** | 35.0 | 23.0 | 24.0 | 25.0 | 85 | 49 | 3.1 | 5.0 | 5.15 | N-E | 7.30 | 26.5 | 26.9 | 27.4 | 28.1 | 29.4 |
| **44** | 34.0 | 22.0 | 24.0 | 26.0 | 86 | 53 | 2.4 | 40.0 | 4.65 | N-E | 5.30 | 25.5 | 25.8 | 26.6 | 27.5 | 28.4 |
| **45** | 32.0 | 22.0 | 24.0 | 25.5 | 90 | 56 | 2.3 | 0 | 4.68 | N-E | 7.31 | 26.0 | 26.0 | 26.6 | 27.0 | 28.0 |
| **46** | 31.0 | 20.0 | 23.5 | 24.5 | 90 | 58 | 1.7 | 14.9 | 4.73 | S-E | 7.32 | 26.8 | 27.0 | 27.2 | 28.3 | 29.2 |
| **47** | 30.0 | 19.0 | 22.5 | 23.5 | 89 | 61 | 2.0 | 33.5 | 4.96 | N-E | 5.40 | 26.7 | 26.7 | 27.4 | 28.0 | 29.2 |
| **48** | 29.0 | 20.5 | 23.5 | 24.0 | 91 | 61 | 1.9 | 157.0 | 5.19 | S-W | 6.21 | 25.0 | 25.2 | 25.5 | 26.2 | 27.3 |
| **49** | 31.0 | 22.0 | 22.5 | 24.0 | 88 | 55 | 2.9 | 149.0 | 5.09 | N-E | 7.00 | 24.5 | 24.5 | 25.1 | 25.7 | 27.0 |
| **50** | 29.0 | 19.5 | 20.5 | 21.5 | 91 | 55 | 3.1 | 154.0 | 4.64 | N-E | 6.30 | 23.8 | 24.5 | 24.9 | 25.5 | 26.5 |
| **51** | 31.0 | 20.5 | 21.4 | 22.0 | 89 | 51 | 4.3 | 0 | 5.00 | N-E | 8.30 | 25.6 | 25.5 | 26.3 | 27.5 | 29.0 |
| **52** | 30.0 | 20.5 | 21.5 | 23.0 | 85 | 55 | 3.1 | 76.0 | 4.53 | N-E | 5.00 | 22.8 | 23.4 | 24.0 | 25.0 | 26.2 |

|  | | | **Weekly Weather Data – 2023** | | | | | | | | | |  | | | |
| --- | --- | --- | --- | --- | --- | --- | --- | --- | --- | --- | --- | --- | --- | --- | --- | --- |
|  | | | | | | | | | | | | | | | | |
| **S.No** | **Max** | **Min** | **Wet** | **Dry** | **RH 07.00am & 14.00pm** | | **Evop** | **Rain** | **W-S** | **W-D** | **Sun** | **Soil Temperature** | | | | |
|  |  |  |  |  |  |  |  |  |  |  |  | **5cm** | **10cm** | **15cm** | **20cm** | **30cm** |
| **1** | 29.0 | 19.0 | 16.0 | 17.5 | 89 | 61 | 5.0 | - | 5.20 | N-W | 6.00 |  |  |  |  |  |
| **2** | 29.0 | 16.0 | 17.0 | 19.0 | 88 | 52 | 5.1 | - | 5.12 | N-E | 6.40 |  |  |  |  |  |
| **3** | 29.5 | 15.5 | 18.0 | 19.5 | 88 | 56 | 5.5 | - | 4.76 | N-E | 7.00 |  |  |  |  |  |
| **4** | 29.5 | 17.0 | 18.0 | 20.0 | 87 | 57 | 4.8 | - | 4.75 | N-E | 6.15 |  |  |  |  |  |
| **5** | 30.0 | 18.0 | 17.0 | 18.5 | 89 | 52 | 5.2 | - | 5.25 | N-E | 7.00 |  |  |  |  |  |
| **6** | 31.5 | 17.5 | 18.0 | 21.0 | 90 | 45 | 5.3 | - | 5.46 | N-E | 8.50 |  |  |  |  |  |
| **7** | 32.0 | 16.0 | 18.5 | 20.5 | 87 | 38 | 4.9 | - | 5.89 | N-E | 8.30 |  |  |  |  |  |
| **8** | 32.0 | 17.0 | 19.0 | 22.0 | 87 | 40 | 4.8 | - | 5.56 | N-E | 8.30 |  |  |  |  |  |
| **9** | 33.0 | 16.5 | 21.0 | 23.5 | 88 | 46 | 5.5 | - | 5.36 | N-E | 7.15 |  |  |  |  |  |
| **10** | 32.0 | 17.0 | 20.5 | 23.0 | 88 | 38 | 5.7 | - | 5.75 | N-E | 7.10 |  |  |  |  |  |
| **11** | 33.0 | 20.0 | 20.5 | 24.0 | 88 | 39 | 5.3 | - | 5.47 | N-E | 7.20 |  |  |  |  |  |
| **12** | 33.0 | 19.5 | 19.0 | 24.5 | 85 | 40 | 5.7 | - | 5.75 | N-E | 7.10 |  |  |  |  |  |
| **13** | 33.5 | 19.0 | 22.0 | 24.5 | 84 | 48 | 6.0 | - | 6.16 | N-E | 7.15 |  |  |  |  |  |
| **14** | 36.0 | 26.5 | 21.0 | 23.0 | 85 | 51 | 6.0 | - | 5.71 | N-E | 8.15 |  |  |  |  |  |
| **15** | 38.0 | 26.5 | 22.5 | 25.0 | 81 | 38 | 5.8 | - | 5.81 | N-E | 8.15 |  |  |  |  |  |
| **16** | 38.0 | 24.5 | 20.0 | 14.0 | 78 | 38 | 6.2 | 16.0 | 5.65 | N-E | 8.30 |  |  |  |  |  |
| **17** | 36.0 | 26.0 | 24.0 | 27.5 | 86 | 51 | 4.5 | 5.8 | 6.20 | N-E | 9.10 |  |  |  |  |  |
| **18** | 35 | 25.5 | 23.5 | 26.5 | 84 | 60 | 5.6 | - | 5.45 | N-E | 5.30 |  |  |  |  |  |
| **19** | 35.5 | 26.0 | 26.0 | 28.5 | 89 | 56 | 5.8 | 83.4 | 6.36 | N-E | 7.00 |  |  |  |  |  |
| **20** | 39.0 | 28.0 | 25.5 | 28.0 | 82 | 49 | 6.4 | 28.0 | 5.35 | N-E | 8.30 |  |  |  |  |  |
| **21** | 37.5 | 27.5 | 26.0 | 29.0 | 82 | 47 | 5.4 | - | 6.18 | N-E | 7.40 |  |  |  |  |  |
| **22** | 39.0 | 27.5 | 27.0 | 30.0 | 78 | 46 | 5.6 | - | 5.14 | N-E | 7.30 | 27.7 | 28.2 | 29.5 | 29.7 | 31.3 |
| **23** | 39.5 | 29.5 | 30.0 | 32.0 | 78 | 46 | 5.6 | 18.0 | 4.34 | S-E | 7.50 | 30.2 | 30.9 | 31.3 | 32.0 | 32.8 |
| **24** | 38.5 | 28.5 | 28.0 | 30.0 | 78 | 46 | 5.9 | 58.0 | 5.27 | N-E | 4.30 | 28.9 | 29.0 | 30.0 | 31.0 | 32.5 |
| **25** | 32.5 | 28.0 | 24.5 | 26.5 | 88 | 55 | 1.9 | 103.0 | 8.30 | S-E | 1.30 | 25.7 | 26.5 | 27.0 | 27.4 | 28.2 |
| **26** | 34.5 | 27.0 | 25.0 | 28.0 | 79 | 52 | 4.9 | - | 6.77 | S-E | 5.20 | 28.3 | 28.5 | 29.2 | 29.9 | 30.7 |
| **27** | 35.0 | 29.0 | 25.5 | 28.5 | 76 | 44 | 6.3 | - | 6.17 | N-E | 5.00 | 29.0 | 29.4 | 29.8 | 30.5 | 32.2 |
| **28** | 34.0 | 28.0 | 25.0 | 26.5 | 85 | 57 | 3.1 | 126.1 | 7.60 | N-E | 5.20 | 26.0 | 26.5 | 27.6 | 27.7 | 28.5 |
| **29** | 35.0 | 29.0 | 26.0 | 28.0 | 73 | 42 | 6.2 | - | 5.81 | N-E | 4.00 | 28.1 | 28.1 | 28.7 | 29.3 | 30.0 |
| **30** | 33.5 | 29.0 | 24.5 | 26.5 | 85 | 53 | 4.4 | 9.0 | 6.35 | N-E | 7.50 | 26.1 | 26.2 | 26.9 | 27.1 | 28.4 |
| **31** | 35.0 | 30.0 | 26.0 | 28.5 | 77 | 43 | 4.6 | 7.0 | 6.58 | N-E | 6.10 | 28.7 | 29.2 | 29.6 | 30.2 | 32.0 |
| **32** | 35.5 | 29.0 | 26.0 | 28.0 | 84 | 45 | 4.3 | 16.5 | 6.94 | N-E | 6.20 | 27.2 | 28.5 | 29.2 | 29.7 | 31.0 |
| **33** | 36.0 | 29.0 | 25.5 | 28.0 | 81 | 46 | 3.8 | 25.0 | 6.87 | N-E | 5.40 | 25.9 | 26.2 | 27.0 | 27.7 | 28.4 |
| **34** | 35.5 | 29.0 | 26.0 | 27.0 | 83 | 44 | 4.0 | 16.8 | 7.42 | N-E | 5.40 | 27.3 | 28.2 | 29.0 | 29.8 | 31.4 |
| **35** | 35.5 | 30.0 | 25.0 | 26.5 | 85 | 45 | 3.2 | 37.0 | 7.06 | N-E | 7.00 | 26.2 | 26.4 | 27.4 | 28.0 | 29.2 |
| **36** | 33.0 | 30.0 | 25.5 | 27.5 | 87 | 41 | 3.7 | 63.6 | 7.76 | N-E | 5.00 | 26.1 | 26.2 | 26.8 | 27.2 | 28.5 |
| **37** | 35.0 | 30.0 | 26.5 | 29.0 | 80 | 41 | 4.5 | - | 6.72 | S-E | 5.40 | 28.0 | 28.2 | 29.0 | 29.7 | 30.7 |
| **38** | 34.5 | 29.0 | 25.5 | 27.5 | 85 | 42 | 5.0 | 14.5 | 4.50 | S-E | 5.50 | 26.1 | 26.3 | 27.0 | 27.8 | 29.0 |
| **39** | 34.5 | 28.0 | 24.0 | 26.5 | 90 | 45 | 4.3 | 374.0 | 6.57 | S-E | 6.40 | 25.8 | 26.0 | 26.5 | 27.0 | 28.3 |
| **40** | 34.0 | 28.0 | 24.0 | 27.0 | 82 | 42 | 5.9 | 3.0 | 6.46 | N-E | 7.40 | 26.5 | 27.1 | 27.9 | 28.5 | 29.7 |
| **41** | 34.0 | 27.0 | 24.5 | 27.0 | 83 | 42 | 4.3 | 30.0 | 5.90 | N-E | 6.30 | 27.3 | 27.0 | 28.0 | 29.5 | 30.6 |
| **42** | 33.0 | 25.0 | 23.5 | 26.0 | 80 | 43 | 4.5 | - | 5.49 | N-E | 7.10 | 26.2 | 27.3 | 28.3 | 29.2 | 30.0 |
| **43** | 33.0 | 24.0 | 24.5 | 23.0 | 89 | 54 | 4.1 | - | 4.70 | N-E | 5.30 | 25.8 | 26.0 | 26.6 | 27.0 | 28.5 |
| **44** | 32.0 | 24.0 | 24.0 | 25.5 | 89 | 54 | 3.7 | 35.0 | 4.75 | N-E | 5.50 | 25.8 | 26.0 | 26.6 | 27.0 | 28.6 |
| **45** | 32.0 | 25.0 | 23.5 | 25.0 | 90 | 66 | 3.5 | - | 5.14 | N-E | 7.08 | 25.2 | 25.3 | 26.0 | 26.5 | 27.5 |
| **46** | 32.0 | 25.0 | 23.5 | 25.5 | 86 | 59 | 3.7 | 4 | 4.93 | N-E | 5.30 | 24.5 | 25.2 | 25.7 | 26.1 | 27.2 |
| **47** | 31.5 | 24.0 | 24.0 | 25.0 | 88 | 54 | 3.8 | 4.7 | 4.61 | N-E | 6.20 | 24.8 | 25.2 | 25.7 | 26.2 | 27.2 |
| **48** | 30.0 | 22.0 | 23.5 | 25.0 | 90 | 58 | 4.2 | 52.0 | 4.78 | N-E | 6.00 | 24.3 | 24.5 | 24.8 | 25.5 | 26.3 |
| **49** | 30.0 | 20.0 | 24.0 | 25.0 | 86 | 59 | 4.2 | - | 4.59 | N-E | 7.00 | 24.1 | 24.7 | 25.4 | 25.7 | 26.7 |
| **50** | 28.0 | 20.0 | 24.0 | 25.5 | 89 | 59 | 4.7 | - | 4.89 | S-E | 7.50 | 23.8 | 24.3 | 24.8 | 25.2 | 26.7 |
| **51** | 29.0 | 18.0 | 24.0 | 26.0 | 88 | 58 | 4.0 | - | 4.82 | S-E | 6.20 | 23.8 | 24.5 | 25.0 | 25.6 | 27.1 |
| **52** | 30.0 | 20.0 | 20.5 | 23.5 | 87 | 53 | 4.5 | - | 4.74 | N-E | 7.00 | 24.5 | 25.0 | 25.9 | 26.6 | 28.0 |

12 March.2025

Rainfall – 8.5mm
